# Genetic Variants Underlying Risk of Intracranial Aneurysms: Insights from a GWAS in Portugal

**DOI:** 10.1371/journal.pone.0133422

**Published:** 2015-07-17

**Authors:** Patrícia Abrantes, Maria M. Santos, Inês Sousa, Joana M. Xavier, Vânia Francisco, Tiago Krug, João Sobral, Mafalda Matos, Madalena Martins, António Jacinto, Domingos Coiteiro, Sofia A. Oliveira

**Affiliations:** 1 Instituto Medicina Molecular, Faculdade de Medicina, Universidade de Lisboa, Lisboa, Portugal; 2 Instituto Gulbenkian de Ciência, Oeiras, Portugal; 3 Serviço de Neurocirurgia, Hospital de Santa Maria, Lisboa, Portugal; 4 Centro de Estudos de Doenças Crónicas (CEDOC), Faculdade de Ciências Médicas, Universidade Nova de Lisboa, Lisboa, Portugal; St Michael's Hospital, University of Toronto, CANADA

## Abstract

Subarachnoid hemorrhage (SAH) is a life-threatening event that most frequently leads to severe disability and death. Its most frequent cause is the rupture of a saccular intracranial aneurysm (IA), which is a blood vessel dilation caused by disease or weakening of the vessel wall. Although the genetic contribution to IA is well established, to date no single gene has been unequivocally identified as responsible for IA formation or rupture. We aimed to identify IA susceptibility genes in the Portuguese population through a pool-based multistage genome-wide association study. Replicate pools were allelotyped in triplicate in a discovery dataset (100 IA cases and 92 gender-matched controls) using the Affymetrix Human SNP Array 6.0. Top SNPs (absolute value of the relative allele score difference between cases and controls |RAS_diff_|≥13.0%) were selected for technical validation by individual genotyping in the discovery dataset. From the 101 SNPs successfully genotyped, 99 SNPs were nominally associated with IA. Replication of technically validated SNPs was conducted in an independent replication dataset (100 Portuguese IA cases and 407 controls). rs4667622 (between *UBR3* and *MYO3B*), rs6599001 (between *SCN11A* and *WDR48*), rs3932338 (214 kilobases downstream of *PRDM9*), and rs10943471 (96 kilobases upstream of *HTR1B*) were associated with IA (unadjusted allelic chi-square tests) in the datasets tested (discovery: 6.84E-04≤*P*≤1.92E-02, replication: 2.66E-04≤*P*≤2.28E-02, and combined datasets: 6.05E-05≤*P*≤5.50E-04). Additionally, we confirmed the known association with IA of rs1333040 at the 9p21.3 genomic region, thus validating our dataset. These novel findings in the Portuguese population warrant further replication in additional independent studies, and provide additional candidates to more comprehensively understand IA etiopathogenesis.

## Introduction

Approximately 2% of the general population has undetected intracranial aneurysms [1], although only a small fraction of those will experience aneurysm rupture. Of those who present with subarachnoid hemorrhage, nearly 50% will die and a significant percentage is left with important neurological deficits [2]. The great majority of IAs are asymptomatic before presenting with the potentially devastating event of SAH. Treatment after bleeding, either through endovascular or microsurgical procedures, is also associated with higher morbidity and mortality [3–4]. On the other hand, those patients carrying aneurysms that will never rupture should not be subjected to the risks involved with aneurysmal repair. Therefore, diagnosis before rupture and accurate biomarkers to predict the risk of rupture are of paramount importance to the IA patient’s survival and quality of life. Despite recent advances in molecular techniques, diagnosis of IA still relies solely on imaging methods and the mechanisms of IA initiation, progression, and rupture are still poorly understood.

Intracranial aneurysms are thought to result from the interplay of environmental and genetic factors. Hypertension, smoking and alcohol consumption are major determinants of IA risk [5–6] showing a strong interaction with each other but also with non-modifiable IA risk factors such as ethnicity, gender, age, IA localization and size, and genetic background. Genetic contribution to IA is supported by studies on monozygotic twins, familial occurrence, and also by its co-morbidity with other genetic diseases such as adult polycystic kidney disease [7–8]. Nevertheless, progress in finding IA genes and the disease-associated alleles has proven difficult and to date no single gene has been unequivocally identified as responsible for IA formation or rupture.

Genome-wide association studies (GWAS) represent the gold-standard approach for uncovering novel genetic risk variants and have brought novel insights into the biological and genetic underpinnings of many complex traits. To date, several GWAS have been conducted in IA. Bilguvar et al. [9] found associations at 2q33, 8q11 and 9p21 genomic regions in Finnish, Dutch and Japanese cohorts. A follow-up study with additional European and Japanese cases [10] identified three new loci (10q24.32, 13q13.1 and 18q11.2) and replicated two of those previously found (8q11 and 9p21). These five risk loci explain only up to 5% of the familial risk of intracranial aneurysms [11]. Akiyama et al. [12] found 4 loci (1q23.1, 3p25.2, 7p21.2 and 9q31.3) associated with IA in Japanese population but none of them reached genome-wide significance. Low et al. [13] reported a new locus (near *EDNRA*) on chromosome 4q31.22 and replicated the 9p21.3 region in the Japanese population. Foroud et al. [14] strengthened the association of the *CDKN2BAS* locus at 9p21.3 with IA in a European population, and a follow-up of this GWAS in a larger dataset of white ancestry [15] detected a novel association of rs10230207 near *HDAC9* (histone deacetylase 9), a gene previously associated with ischemic stroke [16–17]. Finally, a GWAS in the Finns [18] established the association of five loci (2q23.3, 2q33.1, 5q31.1, 6q24.2, and 9p21.3) with saccular IAs. 9p21.3 is the only locus consistently replicated across different studies [9–10, 13–15, 18–19], reinforcing its likely involvement in IA etiology.

In the present paper, we use a pool-based multistage GWAS approach as a cost-effective, time- and labour-saving alternative to identify novel susceptibility loci for intracranial aneurysms in a Portuguese dataset. Pool-based GWAS is an efficient strategy to reduce the high costs associated with individual-based GWAS but is also effective at identifying major genetic contributions to disease. This validated approach replaces individual genotyping of every person by allelotyping of pools of individuals. Numerous studies have followed this approach [20–21] and true effectiveness of the technique was successfully confirmed by the identification of previously published as well as novel genetic susceptibility loci [22–24]. We hereby describe the first IA GWAS performed in a southern European population, in which we perform first a discovery phase, followed by a technical validation stage, and then finally an independent replication and combined analysis.

## Materials and Methods

### Subjects

All cases were enrolled in the Neurosurgery Department at *Hospital de Santa Maria* (HSM) in Lisbon and were diagnosed with saccular intracranial aneurysm by computerized tomography angiogram, magnetic resonance angiogram, or digital subtraction angiography, confirmed at surgery when applicable. Patients with known heritable diseases (including Ehlers-Danlos syndrome type IV, Marfan syndrome, Moya-Moya syndrome and autosomal-dominant polycystic kidney disease) were excluded from the study. Rupture of an aneurysm was defined by the identification of SAH through computed tomography or lumbar puncture from a proven aneurysm.

Control subjects were collected at the *Instituto Português do Sangue e da Transplantação* (IPST) and the *Universidade Nova Atena*, and requested from Biobanco-IMM, Lisbon Academic Medical Center, Lisbon, Portugal. Controls were screened for not having a known personal history of any stroke or IA, and no known family history of IA or SAH.

All participants are of European descent (Portuguese origin) and provided written informed consent. The Hospital de Santa Maria’s ethical committee approved the study protocol and the study was conducted according to the Declaration of Helsinki. The clinical and demographic features of the study participants were obtained by medical interview at the time of blood sampling, and by inspection of medical records.

### Construction of DNA pools

Genomic DNA was extracted from whole blood using QIAamp DNA Blood Maxi Kit (QIAGEN). The concentration of extracted DNA was determined by Nanodrop using absorbance reading at 260nm. DNA samples from 106 IA cases and 101 control samples matched for age and gender (*P* = 5.60E-02 and 6.50E-02, respectively) were diluted to an approximate concentration of 75ng/μL and their concentration was then quantified in triplicate by fluorimetry (Quant-iT PicoGreen dsDNA Assay Kit, Invitrogen) using a PerkinElmer top fluorescence reader (PerkinElmer, Inc, Waltham, USA). Samples with >2 of the sample standard deviation (SD) from the median volume to be pooled for each sample (6 cases and 9 controls) were not included in the respective pool. The 100 cases and 92 controls passing quality control were pooled in equimolar amounts (200ng of each sample) according to their affection status. To minimise pipetting-associated errors, no less than 1.3μl of each sample was added to a pool. Two replicate pools of each group were prepared independently, quantified and diluted to 50ng/μl.

### Genome-wide allelotyping

The Affymetrix Genome-Wide Human SNP Arrays 6.0 (Santa Clara, CA, USA) were processed at the Instituto Gulbenkian de Ciência’s Affymetrix Core Facility following manufacturer’s protocols. Probe-intensity data were directly read from cell-intensity (CEL) files and Relative Allele Scores (RAS) were calculated for each quartet using the SNPMaP package [25]. A RAS corresponds to the ratio of the A probe to the sum of the A and B probes (where A is the major allele and B is the minor allele) and provides a quantitative index correlated to the allele frequency in pooled DNA. RAS distribution of replicate arrays was analysed using box-and-whisker plots.

All copy number variation-related SNPs, as well all the polymorphisms on sex and mitochondrial chromosomes were excluded from further analyses.

Pearson’s correlation coefficient was calculated between the average of the RAS values of cases and controls using the R freeware (http://cran.r-project.org/).

### Individual genotyping

Except for rs1333040, individual samples were genotyped using Sequenom's (San Diego, USA) iPlex assay (primer extension of multiplex products with detection by matrix assisted laser desorption/ionization time-of-flight mass spectrometry) following manufacturer's protocol and detected in a Sequenom MassArray K2 platform. The primer sequences (S1 Table) were designed using Sequenom's MassARRAY Assay Design 3.0 software. Genotyping was performed in the Genomics Unit of the Instituto Gulbenkian de Ciência.

For rs1333040, individuals were genotyped using the TaqMan SNP Genotyping Assay-on-demand C_8766795_10 (Life Technologies, USA), with the TaqMan Genotyping Master Mix on a ViiA 7 Real-Time PCR System.

Extensive quality control was performed using eight HapMap (http://hapmap.ncbi.nlm.nih.gov/) controls of diverse ethnic affiliation, sample duplication within and across plates, Mendelian inheritance check in one large pedigree (without IA), Hardy-Weinberg equilibrium (HWE) in the control group (*P*>0.001), and a minimum of 90% sample and 85% SNP call rates. Genotype determinations were performed blinded to affection status.

### Association analyses

Unpaired Student’s t-tests and χ2 tests were used to compare quantitative and qualitative clinical and demographic data, respectively, between IA cases and controls. The adapted Manhattan plot was created using the R freeware. χ2 tests for HWE in controls and crude allelic association of SNPs with IA risk were performed using Haploview 4.2 [26]. Linkage disequilibrium (LD) plots were performed using Haploview 4.2 and SNAP v2.2 [27]. Haplotype tagging SNPs were identified in Haploview using pairwise tagging only and an r^2^ threshold of 0.8.

Adjusted and unadjusted association analyses between IA and each SNP were performed with logistic regression (additive genetic model) using the SNPassoc v1.4–9 package [28] implemented in R. To adjust the association analyses for relevant confounding factors, hypertension and ever smoking were included as covariates in multivariate logistic regression with backward elimination of risk factors. The interaction *i* among these covariates in regression models was not strong (-0.5<*i*<0.5). Odds ratios (ORs) and their associated 95% confidence intervals (CIs) were uncorrected for confounding variables in the χ2 tests and unadjusted logistic regression, and corrected for covariates in adjusted regression models. Nominally associated SNPs (*P*≤0.05) in the technical validation and independent replication phases were considered eligible for further analyses. A combined analysis of the discovery and replication datasets was performed for the SNPs associated in both phases of the GWAS and for rs1333040.

## Results

### DNA pooling and GWAS discovery phase

To identify genetic variants associated with IA in the Portuguese population, we first performed a GWAS in the discovery dataset using pools of samples. The main demographic and clinical characteristics of the 100 cases with IA and 92 controls constituting the discovery dataset are summarized in Table 1. These patients and controls are gender- but not age-matched (*P* = 5.20E-02 and 4.70E-02, respectively). Similarly to larger datasets [12–14], the average age-at-examination of the IA patients was 53.6±10.9 years and 68.0% were female. 70.0% of patients suffered ruptured aneurysms, 5.0% had a known family history of IA, 55.1% had hypertension, and 52.0% declared having smoking habits at some point during their life.

**Table 1:** General characteristics of the Portuguese discovery and replication datasets.

	Discovery dataset		Replication dataset	
Characteristics	Cases	Controls	Cases	Controls
*N*	100	92	100	407
Gender (*n/N*, % women)	68/100 (68.0)	50/92 (54.3)	73/100 (73.0)	255/407 (62.6)
Age-at-examination (mean ± SD, ys)	53.6 ± 10.9	50.0 ± 14.2	59.6 ± 15.4	52.0 ± 13.5
Hypertension (*n/N*, %)	54/98 (55.1)	22/91 (24.1)	55/96 (57.2)	78/404 (19.3)
Ever smoking (*n/N*, %)	52/100 (52.0)	45/92 (48.9)	42/95 (44.2)	104/390 (26.6)
Alcohol drinking habit (*n/N*, %)	51/100 (51.0)	33/69 (47.8)	44/97 (45.3)	70/316 (39.5)
Family history of IA (*n/N*, %)	5/99 (5.0)	0/90 (0)	2/95 (2.1)	0/59 (0)
SAH (*n/N*, %)	49/70 (70.0)	-	57/82 (69.5)	-
Vasospasm (*n/N*, %)	35/66 (53.0)	-	31/75 (41.3)	-

IA: Intracranial aneurysm; ys: years; SAH: subarachnoid hemorrhage; SD: Standard deviation.

DNA samples from this discovery dataset passed our quality controls for pooling and were mixed in duplicate in equimolar amounts and allelotyped in triplicate on Affymetrix Genome-Wide Human SNP Arrays 6.0 featuring over 906,600 SNPs distributed across the entire genome. Two replicate pools of cases/controls were constructed and each hybridized three times (total of 12 arrays) rather than constructing several smaller pools and hybridising them on single arrays, as this method has been shown to be more powerful [29]. One of the replicate arrays from a case pool exhibited a different RAS distribution from the other arrays (data not shown) and was therefore excluded from the remaining analyses. Average of RAS values over the remaining 5 case and 6 control arrays showed a strong Pearson correlation with each other (r = 0.991), suggesting that technical variability of the method was low.

**Fig 1:**
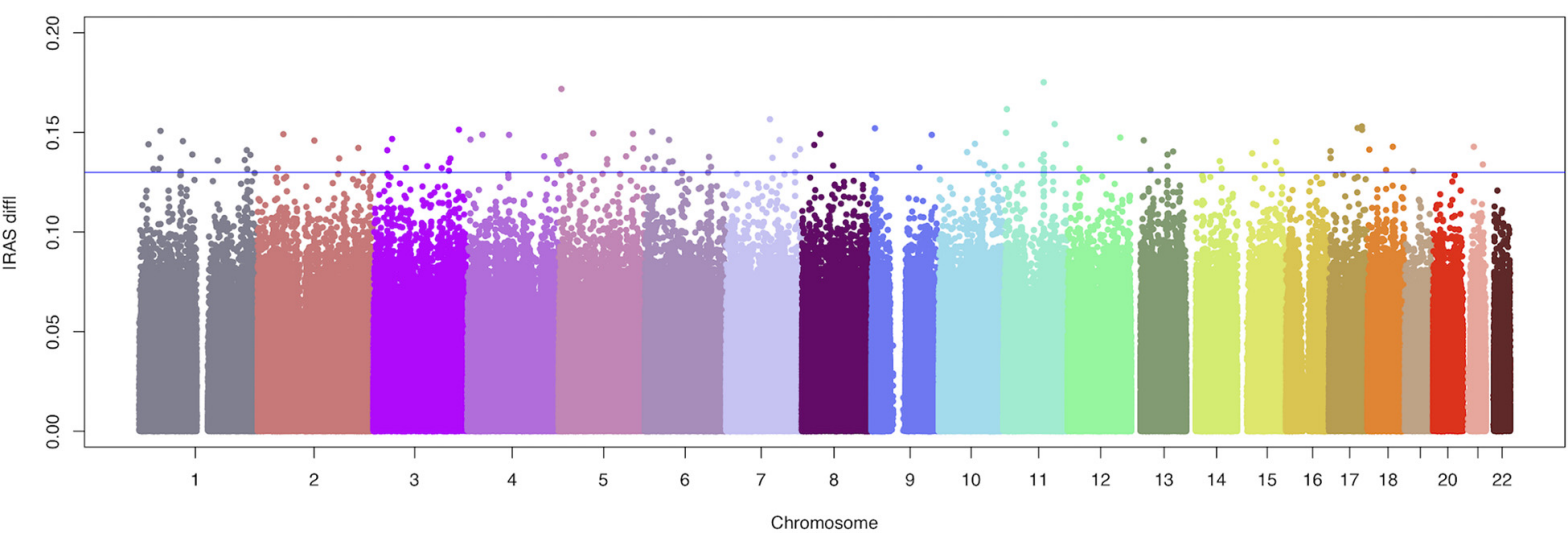
Modified Manhattan plot for the discovery phase of the intracranial aneurysm GWAS using pools. The y-axis represents the absolute value of the relative allele score difference between cases and controls (|RAS_diff_|) for the 868,261 autosomal SNPs allelotyped in 100 Portuguese cases and 92 controls, and the x-axis refers to their chromosomal position. A blue line is drawn at the |RAS_diff_| cutoff of 13.0%.

A total of 868,261 SNPs which are not in copy number variants, mitochondrial or sex chromosomes were further analysed. Fig 1 depicts the absolute value of the RAS difference between cases and controls (|RAS_diff_|) for all the assayed autosomal genetic markers. The polymorphisms were ranked by |RAS_diff_| and the 113 markers with |RAS_diff_|≥13.0% are listed in S2 Table.

The next step in our multistage GWAS was to technically validate the top findings of the pool-based GWAS using a different technique, namely by individually genotyping the top markers in the discovery dataset (100 cases and 92 controls). Out of the 113 SNPs selected for the GWAS technical validation phase, five polymorphisms (rs17053831, rs12991586, rs13355489, rs26118 and rs1922648) were not included in the assay design phase because their minor allele frequency (MAF) in the HapMap CEU samples was low (<0.10) for our dataset to have enough power to detect an association, 2 SNPs (rs1744895 and rs10416963) failed in the assay design step, and 5 markers (rs10834648, rs16977458, rs2949574, rs1357938 and rs10014056) failed in the genotyping quality control (e.g., genotyping errors, low call rate).

From the 101 SNPs successfully genotyped, 99 SNPs (98.0%) were technically validated since they were nominally associated with IA according to an allelic chi-square test (S3 Table). Forty of these markers remain significantly associated with IA after Bonferroni correction for multiple testing (*P*≤4.95E-04).

### Independent replication of SNPs associated in the discovery phase

The third phase in this multistage GWAS is to confirm our positive findings from the discovery phase in an independent replication dataset. The 99 SNPs nominally associated with IA in the discovery dataset were successfully tested for association in a second group of 100 Portuguese IA cases and 407 controls matched for gender (*P* = 5.24E-02) but not for age (*P* = 1.65E-05) (Table 1). As expected for IA risk factors, hypertension and smoking habits were significantly more frequent in IA cases than in controls (57.2% versus 19.3%, *P*<1.00E-04 and 44.2% versus 26.6%, *P* = 8.20E-04, respectively).

Eleven polymorphisms (rs2854108, rs10915933, rs10799384, rs4667622, rs6599001, rs3932338, rs10943471, rs12691433, rs4465006, rs7865885, and rs7048859) were also nominally associated in the replication dataset using an allelic chi-square test (S3 Table). Association analysis of these 11 SNPs in the combined discovery and replication dataset using the same statistical test strengthened the associations detected at four SNPs: rs4667622, rs6599001, rs3932338 and rs10943471 (Table 2).

**Table 2:** IA association results for the SNPs associated in the discovery, replication and combined datasets.

SNP	Chr.	Neighboring genes (distance)	Location	Dataset	Allele	F_cases_	F_controls_	*P*	*P*_*adj*_	OR[95% CI]
rs4667622	2	*UBR3* (63 kb)	Intergenic	Discovery	G	0.593	0.418	**6.84E-04**	**3.67E-04**	2.22[1.41–3.57]
		*MYO3B* (30 kb)		Replication		0.592	0.482	**9.90E-03**	**2.13E-02**	1.54[1.06–2.22]
				Combined		0.592	0.469	**7.13E-05**	**4.00E-05**	1.75[1.33–2.33]
rs6599001	3	*SCN11A* (82 kb)	Intergenic	Discovery	T	0.803	0.901	**7.48E-03**	**1.49E-02**	0.46[0.24–0.88]
		*WDR48* (19 kb)		Replication		0.811	0.886	**5.10E-03**	**5.48E-03**	0.49[0.30–0.81]
				Combined		0.807	0.889	**6.05E-05**	**2.20E-04**	0.50[0.35–0.72]
rs3932338	5	*PRDM9* (214 kb)	Intergenic	Discovery	G	0.333	0.451	**1.92E-02**	1.33E-01	-
				Replication		0.242	0.383	**2.66E-04**	**2.50E-04**	0.48[0.32–0.72]
				Combined		0.288	0.395	**2.02E-04**	**1.29E-03**	0.63[0.48–0.84]
rs10943471	6	*HTR1B* (96 kb)	Intergenic	Discovery	A	0.753	0.879	**1.56E-03**	**5.10E-04**	0.35[0.19–0.66]
				Replication		0.750	0.822	**2.28E-02**	**2.94E-02**	0.63[0.42–0.95]
				Combined		0.751	0.832	**5.50E-04**	**3.21E-04**	0.55[0.40–0.76]

Odds ratios (OR) and 95% confidence intervals (CI) are relative to the allele on the forward strand of the human genome reference sequence and are only shown for those markers with nominally significant associations (*P*_*adj*_≤5.00E-02). Significant associations are highlighted in bold.

SNP: Single nucleotide polymorphism; kb: Kilobase pairs; F_cases_ and F_controls_: Allele frequency in cases and controls, respectively; *P*: Allelic qui-square P-value. *P*_*adj*_: *P*-values from logistic regression using log-additive model are adjusted for hypertension in the discovery dataset, for hypertension and smoking habits in the replication and combined datasets; *UBR3*: ubiquitin protein ligase E3 component n-recognin 3 gene; *MYO3B*: myosin IIIB gene; *SCN11A*: sodium channel, voltage-gated, type XI, alpha subunit gene; *WDR48*: WD repeat domain 48 gene; *PRDM9*: PR domain containing 9 gene; *HTR1B*: 5-hydroxytryptamine (serotonin) receptor 1B gene.

SNPs rs4667622 (*P* = 7.13E-05, OR_G_[95% CI] = 1.65[1.29–2.11]) and rs6599001 (*P* = 6.05E-05, OR_T_[95% CI] = 0.52[0.38–0.72]) were the most significant in the combined dataset. rs4667622 is located on chromosome 2q31.1 within a regulatory region (63 kilobases downstream) of the ubiquitin protein ligase E3 component n-recognin 3 gene (*UBR3*) and (30 kilobases upstream) of the myosin IIIB gene (*MYO3B*) and rs6599001 maps to chromosome 3p22.2, between the *SCN11A* (sodium channel, voltage-gated, type XI, alpha subunit) and *WDR48* (WD repeat domain 48) genes, although closest to *WDR48*. The third most significant genetic association was found with rs3932338 (*P* = 2.02E-04, OR_G_[95% CI] = 0.62[0.48–0.80]) which is located on chromosome 5p14.2, in a gene desert 214 kilobases downstream of *PRDM9* (PR domain containing 9). Finally, the last SNP associated in all datasets tested was rs10943471 (*P* = 5.50E-04, OR_A_[95% CI] = 0.61[0.46–0.81]) which is located on chromosome 6q14.1 in an intergenic region 96 kilobases upstream of *HTR1B* (5-hydroxytryptamine (serotonin) receptor 1B).

To account and correct for unequal frequency of risk factors in cases and controls in the different datasets, we performed association analyses adjusted for the relevant co-variates in each dataset (hypertension in the discovery dataset, hypertension and smoking status in the replication and combined datasets). These four top SNPs were also associated with IA in adjusted tests in all datasets tested (4.00E-05≤*P*_*adj*_≤2.94E-02), with the exception of rs3932338 which was only marginally associated (*P*_*adj*_ = 1.33E-01) in the adjusted analysis in the discovery dataset (Table 2).

To determine if these four intergenic SNPs may be tagging neighboring intragenic variants, the patterns of linkage disequilibrium in their genomic regions were investigated in the CEU population panel of the 1000 Genome Project. rs4667622, rs3932338 and rs10943471 are in strong LD (r^2^≥0.8) with other intergenic SNPs (S1 Fig), while rs6599001 is in strong LD with 93 SNPs spanning over 293 kilobases (from rs4553926 through rs57495703) and 6 genes (*SCN11A*, *WDR48*, *GORASP1*, *TTC21A*, *AXUD1* and *XIRP*). Ninety-one of these SNPs are either intronic or intergenic, and the remaining two are in the 5’-untranslated region of *TTC21A* (rs28362645, r^2^ = 0.84) and *AXUD1* (rs13084580, r^2^ = 0.92). Furthermore, since rs6599001 has been shown to be a *cis*-regulatory variant associated with a strong eQTL (*P* = 9.32E-37, personal communication from Dr. F. Cambien) for *WDR48* expression in monocytes [30], we analyzed in more detail the LD structure in this locus (S2 Fig). Within the genomic region extending from rs6599001 until the end of *WDR48* (chromosome 3: 39049155 to 39113844 bp on the NCBI B36 assembly), five haplotype tagging SNPs can be identified using HapMap data release 27 (phaseII+III, February 2009): rs6599001, rs4676481, rs7630022, rs12636980, and rs3732377. Since these last four polymorphisms had |RAS_diff_|<8.4% they were not further individually genotyped.

Since rs1333040 at the 9p21 locus has been consistently associated with IA [9–10, 13, 19] but it was not directly tested through our GWAS as it is not represented in the arrays used, its association with IA in our Portuguese datasets was independently assessed. rs1333040 revealed a trend for association in the discovery (*P* = 1.37E-01 and *P*_*adj*_ = 1.25E-01) and replication (*P* = 1.34E-01 and *P*_*adj*_ = 1.43E-01) datasets, but was nominally associated in the combined dataset (allelic chi-square test: *P* = 2.63E-02, OR_T_[95% CI] = 1.36[1.04–1.77]; adjusted log-additive model: *P*_*adj*_ = 1.93E-02, OR_T_[95% CI] = 1.41[1.05–1.89]). Finally, other SNPs GWAS-associated with IA until December 2014 [9–10, 12–15, 18] were not individually genotyped since their |RAS_diff_| (or of a proxy SNP in strong linkage disequilibrium) ranged from 0.7% to 8.4% (S4 Table), well below the 13.0% follow-up cutoff established in this study.

## Discussion

To identify common genetic determinants increasing the susceptibility to IA in the Portuguese population, we conducted a multistage pool-based GWAS including a discovery phase, a technical validation step and an independent replication. This approach was validated by the technical replication of the associations detected at 98% of the SNPs tested, and the replication of the well-established association of rs1333040 at the 9p21 locus with IA. Novel associations of rs4667622, rs6599001, rs3932338 and rs10943471 with IA were detected in the discovery, replication and combined datasets, and these associations were several orders of magnitude stronger than that of rs1333040.

rs4667622 is located 63 kilobases downstream of *UBR3* and 30 kilobases upstream of *MYO3B*. MYO3B belongs to the class III myosins which are actin-based motors with amino-terminal kinase domains and is mostly expressed in the retina, kidney, and testis [31]. Human MYO3B or its orthologues have been associated to such diverse phenotypes as *Mycobacterium bovis* resistance in cattle [32] and to the development of urinary symptoms after radiotherapy for prostate cancer [33]. More interestingly, UBR3 is an E3 ubiquitin-protein ligase belonging to the N-end rule pathway. It leads to the ubiquitination and subsequent degradation of proteins that do carry specific N-terminal residues that are destabilizing according to the N-end rule. *UBR3* (formerly referred to as *ZNF650*) has been associated to neurological phenotypes, namely ischemic stroke [16] and temporal brain volume [34], and therefore its role in IA warrants further investigation.

rs6599001 maps to chromosome 3p22.2 in the putative regulatory region of both the *SCN11A* (sodium channel, voltage-gated, type XI, alpha subunit, 82 kilobases) and the *WDR48* (WD repeat domain 48, 19 kilobases) genes which run in opposite strands. *WDR48* regulates deubiquitinating complexes and may have a role in tumor suppression [35]. Given that a functional link has been established between rs6599001 and *WDR48* [30], we further investigated our first phase GWAS data in this gene but found no strong evidence of association. While the intergenic rs6599001 SNP is likely not causal, it may be in linkage disequilibrium with the causal variant. rs6599001 may also constitute a synthetic association [36] which must be further investigated using high density genotyping/imputation/haplotyping or next-generation sequencing approaches.

rs3932338 is an intergenic variant mapping to a gene desert on chromosome 5p14.2, 214 kilobases downstream of the nearest gene (*PRDM9*). The PR domain-containing 9 protein has a highly variable tandem-repeat zinc finger DNA-binding domain that plays a key role in determining sequence-specific hotspots of meiotic recombination genome wide [37]. Rare PRDM9 allelic forms have been associated with childhood acute lymphoblastic leukaemia [38–39].

The last SNP associated for the first time with IA is rs10943471, located 96 kilobases upstream of *HTR1B*. This gene encodes for a serotonin receptor implicated in changes in vascular tone and in vasoconstriction of cranial arteries [40–41]. Moreover, the HTR1B protein has been localized to the smooth muscle layer as well as the endothelium of human cerebral arteries [42–43]. Notably, rs10943471 is in relatively strong LD (r^2^ = 0.75 and r^2^ = 0.87 in the CEU and JPT HapMap populations, respectively) with rs4706653 (located 118 kilobases upstream of *HTR1B*) that was marginally associated with IA in a GWAS in the Japanese population (*P* = 5.01E-2, personal communication from Dr. H. Nakaoka).

We did not detect evidence of association at previously described top GWAS findings (summarized in the S5 Table), most likely due to the lack of power of the discovery dataset. The limited statistical power of this study, resulting from the relatively small sample size, is expected to be compensated, at least in part, by the homogeneity of the Portuguese population sample. All study participants were ascertained in the Lisbon region and were of self-reported Caucasian ethnicity. Population stratification could not be directly assessed and corrected for in this study since individual genotypes were not available for ancestry informative markers (AIMs). However, among the top 100 most-informative biallelic AIMs that distinguish the northwestern from southeastern European ancestries of European Americans [44], 69 were represented in the Affymetrix Human SNP arrays 6.0 (directly or through another SNP with r^2^ = 1 in HapMap CEU individuals) and had a |RAS_diff_|<8.0% in the discovery dataset.

Since there is no single gold-standard method to prioritize discovery phase results from pool-based GWAS, we used the |RAS_diff_| approach which has been specifically designed for use with Affymetrix microarrays. The |RAS_diff_| is thought to constitute a good proxy for the allelic frequency difference between individually genotyped cases and controls [45–47]. This method appears to be one of the most sensitive methods to pinpoint differences between cases and controls in presence of low technical variation between pools [29, 48], and has previously been successful in identifying risk factors for other complex diseases [24, 45]. The main drawback of this approach is that RAS variation between replicates is not accounted for and may lead to higher rates of false positives and false negatives when variations amongst pools are high. To decrease the error attributed to pool construction (biological error) and to array differences (technical error), we constructed two biological replicates (pools) and performed three technical replicates (arrays), with a high correlation between arrays. Given that statistical tests are not used to rank SNPs in the discovery phase, it is not possible to correct for multiple testing at this stage. Instead, the strategy adopted was to further investigate by individual genotyping and standard statistical testing the SNPs with a |RAS_diff_|>13.0%, an arbitrary but above background threshold in this study. In the technical validation phase, 40 out of the 101 individually genotyped SNPs were significantly associated with IA after Bonferroni correction for multiple testing (*P*≤4.95E-04). However, we did not restrict our independent replication analysis to these 40 SNPs because the relatively small sample size limits the statistical power of this study, and therefore we opted for a more inclusive rather than exclusive strategy.

As observed in most GWAS published to date, the most significant findings typically map to non-coding genomic regions and do not have an evident functional impact [49]. Therefore, to follow up our novel findings, the association of these 4 SNPs must first be assessed in independent datasets, ideally from different geographic backgrounds and ethnicities. Upon validation, bioinformatics approaches may predict the functional consequence of these non-coding variants (e.g. using data from ENCODE, NRCistrome and NIH Roadmap Epigenomics project and tools such as RegulomeDB, HaploReg, and FunciSNP to predict the effect on chromatin structural changes, transcription factor binding, DNA-protein interaction, DNA methylation, histone modifications) and guide the design of targeted *in vitro* or *in vivo* assays [49].

In summary, through this GWAS for intracranial aneurysms performed for the first time in a southern European population, we found evidence of association with IA for 5 SNPs, 4 of which are novel genetic risk factors for IA and rs1333040 that is in a well-established risk locus. These findings warrant further confirmation in other populations to establish their pathogenic role in IA formation.

## Supporting Information

S1 Fig Regional linkage disequilibrium (LD) plots for rs4667622 (A), rs6599001 (B), rs3932338 (C), and rs10943471 (D). (DOCX)

S2 Fig Pairwise linkage disequilibrium (LD) plot for *WDR48* SNPs. (DOCX)

S1 Table Primer sequences used to genotype 101 single nucleotide polymorphisms (SNPs) in the technical validation phase. (DOCX)

S2 Table 113 single nucleotide polymorphisms (SNPs) with |RAS_diff_|≥13.0% in the pool-based genome-wide association study for intracranial aneurysms. (DOCX)

S3 Table Association results of the single nucleotide polymorphisms (SNPs) tested in the technical validation and independent replication phases. (DOCX)

S4 Table |RAS_diff_| of single nucleotide polymorphisms (SNPs) GWAS-associated with intracranial aneurysms (IA) before December 2014. (DOCX)

S5 Table Top findings of genome-wide association studies for intracranial aneurysms. (DOCX)
