# Supplementary material for: Genetic Variants Underlying Risk of Intracranial Aneurysms: Insights from a GWAS in Portugal
Source: PLoS One. 2015 Jul 17;10(7):e0133422. doi: 10.1371/journal.pone.0133422 (PMC4505843; doi:10.1371/journal.pone.0133422)
Supplement: S1 Fig — (DOCX) [file pone.0133422.s001.docx]

a

b

c

d

**S1 Figure.** **Regional linkage disequilibrium (LD) plots for rs4667622 (A), rs6599001 (B), rs3932338 (C), and rs10943471 (D)**. The pairwise LD (r^2^) between the SNP of interest and surrounding variants and the estimated recombination rate are plotted as a function of genomic position. These plots were constructed by SNAP (SNP Annotation and Proxy Search, http://www.broadinstitute.org/mpg/snap/ldplot.php) using the CEU population panel in the 1000 Genome Project (1000GP) Pilot 1 data and a 250 kilobases (kb) distance limit on each side. The horizontal dashed line is drawn at the r^2^=0.80, and the vertical dashed lines indicate the genomic region encompassing SNPs in strong LD (r^2^≥0.80) with the variant of interest.
