# Supplementary material for: Genetic Variants Underlying Risk of Intracranial Aneurysms: Insights from a GWAS in Portugal
Source: PLoS One. 2015 Jul 17;10(7):e0133422. doi: 10.1371/journal.pone.0133422 (PMC4505843; doi:10.1371/journal.pone.0133422)
Supplement: S2 Fig — (DOCX) [file pone.0133422.s002.docx]

**S2 Figure. Pairwise linkage disequilibrium (LD) plot for *WDR48* SNPs.** This plot was constructed using Haploview and depicts the pairwise r^2^ values for SNPs with minor allele frequency greater than 0.05 in CEU HapMap population and in the genomic region from rs6599001 until the end of *WDR48* (chromosome 3: 39049155 to 39113844 bp on the NCBI B36 assembly). Pairwise r^2^ values were calculated using HapMap data release 27 (phaseII+III, February 2009) and are coded with a white-to-black gradient shading proportional to the magnitude of LD (r^2^=0: white; 0<r^2^<1: shades of grey; r^2^=1: black). Haplotype tagging SNPs were identified in Haploview (using pairwise tagging only and an r^2^ threshold of 0.80) and their names are circled.
