# Supplementary material for: Genetic Variants Underlying Risk of Intracranial Aneurysms: Insights from a GWAS in Portugal
Source: PLoS One. 2015 Jul 17;10(7):e0133422. doi: 10.1371/journal.pone.0133422 (PMC4505843; doi:10.1371/journal.pone.0133422)
Supplement: S1 Table — (DOCX) [file pone.0133422.s003.docx]

**S1 Table. Primer sequences used to genotype 101 single nucleotide polymorphisms (SNPs) in the technical validation phase**

| **SNP** | **PCR primer 1** | **PCR primer 2** | **Extension primer** |
| --- | --- | --- | --- |
| rs542892 | ACGTTGGATGGGGTTTTTACATGGCCCCTT | ACGTTGGATGCTGGCCCACTAGGTATCTTG | TGGCCCCTTTCCATC |
| rs7865885 | ACGTTGGATGTCCTCTCCCTACTCTTCTTC | ACGTTGGATGGGTTGGAGCCTTTTTCACAG | CAGTGAGTTACCCACA |
| rs7463038 | ACGTTGGATGCATAAGGAGTACTCAGCTGG | ACGTTGGATGCGGCCACACAAGACGTTTTT | ACTCAGCTGGGTTCAC |
| rs10732827 | ACGTTGGATGTTTCTCTGGACCTACACTTC | ACGTTGGATGCCTCTTTTGCAGACATGTGG | ACCTACACTTCATCCCT |
| rs270706 | ACGTTGGATGGAACTGGCAAAAAGGGAACG | ACGTTGGATGCATTTTCTCTTTTTCCCCTC | GCAGAGCGCTTTCAAGA |
| rs4667622 | ACGTTGGATGATCAGCAGAGCCATCCATTC | ACGTTGGATGAAATCTCAGTGGCCTATAGC | AGCCATCCATTCAACCTA |
| rs13135261 | ACGTTGGATGATGAGAAATCAGCGGAAGAC | ACGTTGGATGTGGCCTTGAATTTCCAATCC | GGGGAATACATGGGACCT |
| rs209573 | ACGTTGGATGATCATCATCCAAAGCCACCG | ACGTTGGATGAAACACAAGCCAGATGGCAG | CCCCGCCACCGTTGCTACA |
| rs2837636 | ACGTTGGATGGATATCAGAAGGTACTTCCC | ACGTTGGATGAAAGGTTTATTTGGAGCGGG | GGGATGACTCCTACTAATC |
| rs652264 | ACGTTGGATGGCTGCCACTTCTCAAGGATG | ACGTTGGATGACATGTTGTTACCCCAAGGC | ACAGCTTGCTTGATAATAG |
| rs4918006 | ACGTTGGATGGCAGAATGGTGAGCCAATTA | ACGTTGGATGCTTGTGTTAGTCTTCTCGTG | TACCCAGCCTCAGTTATTTC |
| rs2274758 | ACGTTGGATGTTGTCTCCTGAATGCATGCC | ACGTTGGATGCACCAAGGGTCACATTTTCC | AGGATTGCTCTGAGGGCTAT |
| rs6782003 | ACGTTGGATGCAAGGTGAAGATTTAGGGCG | ACGTTGGATGGGAGACTGAGTCTCTATCTG | TTAATTCCAGCCACTACTCTT |
| rs11072994 | ACGTTGGATGTCAAGACCTCAGCAGAATCC | ACGTTGGATGGCAGATAATAGGTGCTCAGG | AGGGATTATTCACATGCTTCC |
| rs10457678 | ACGTTGGATGGGTTTGAATGTGTCACCTCC | ACGTTGGATGTCTCTTAAAGGCCCAACCTC | GGGGTTGAACCTTAATGGCCAA |
| rs1866970 | ACGTTGGATGCCCTTGGACTTCCCACTTAG | ACGTTGGATGTGGTTGCAAGTGACAGAAAC | TTGTACTTCCCACTTAGATGAGT |
| rs7224497 | ACGTTGGATGGGTATCTGCTTACCTAAGAC | ACGTTGGATGCAGGTGGGTTTGGTGGATAC | AACTGTATATTCAATGAAACAGCG |
| rs1499901 | ACGTTGGATGTCATTTCCTGGAGTCCTTGC | ACGTTGGATGGATTTTATTGACTTTGGGAC | GATGGCCAAGATGTTAAATCCTAT |
| rs1005318 | ACGTTGGATGTCATGAGGTGGTCACCTTTC | ACGTTGGATGGAGAAGAAGGCCTGATCAAC | GGACCCTTTCTGGTCTTCTCTCAAT |
| rs6592222 | ACGTTGGATGCACAAGCCTACATGCCTATC | ACGTTGGATGTATGTAGACCAAGCTTTGGG | TCTCCATGCCTATCTGCGGATATCT |
| rs589658 | ACGTTGGATGTTGTACCGAGCAACCAAGAG | ACGTTGGATGAAGGGATGTCACAGTTGTGG | AGGATTGAATGCTTGTGCATATAAA |
| rs7157956 | ACGTTGGATGCGTGAAGTTGAACTACAGGC | ACGTTGGATGTGTTAGGAAGCTGGTCTCTG | GTGGGTTGAAAATGGATTGCTCTAC |
| rs10006104 | ACGTTGGATGGTCATACAGCCATGTATATGC | ACGTTGGATGACATATGCACACCAAAGCAC | CAGCCATGTATATGCATATTAACGTA |
| rs668001 | ACGTTGGATGAACCAGTTTCTTCCCTTGAG | ACGTTGGATGGATTATAGGCATGTGCCACC | GGCCCTTGAGTTATAATCATGCTTAA |
| rs3934723 | ACGTTGGATGGAGTTAGATTTCCAATCCTC | ACGTTGGATGAAGAAGAGCCTCCATTCTCC | GAGCTTTCCAATCCTCTTTCTATCTCT |
| rs9500325 | ACGTTGGATGGACATTATTTGAGCCCCAGC | ACGTTGGATGCGAAATAACCTCATAACTGG | CCCCGTATTTGAGCCCCAGCTCTAGAC |
| rs6864279 | ACGTTGGATGCATACCATGCTCTGGAGTAG | ACGTTGGATGGCTATTAAGGAAAACTACAGG | GAAGACCATGCTCTGGAGTAGACACAT |
| rs10183045 | ACGTTGGATGAGGCCCACATAATATCTCAG | ACGTTGGATGTGCAAGTAGGAAACCGGAAG | CCTCTCTCAGATCAAATGCCTACTGTTG |
| rs932797 | ACGTTGGATGGATCCAAAGCCCTGAGTAAC | ACGTTGGATGCTGTCTACTAACTAAATGC | CAACGAAGATGATGAAACAGATTATTAC |
| rs11756174 | ACGTTGGATGTGCTGTAACTACCTCACCAC | ACGTTGGATGAGAGCACATTAACCCCTCAG | CCCCCTAACTACCTCACCACATTATTAAA |
| rs17516032 | ACGTTGGATGTGAAAATGCCTTCCCCCAAG | ACGTTGGATGGCTGTGGGAATTTGGATTTG | CCCAAGCGGTTCTTA |
| rs10915933 | ACGTTGGATGTCTTTGGGTTGGAGGAAGAG | ACGTTGGATGCAGCAACAAAAGACGGACAG | CTGAAAGGGGACTCCA |
| rs9857513 | ACGTTGGATGTGATTGATCCCACCACACAG | ACGTTGGATGAACTGGGGACTACTAGATGG | CCACCACACAGGTACTC |
| rs12691433 | ACGTTGGATGAAACCAAAAAGGCCTTGACC | ACGTTGGATGCCCTCTGACTCAAAAGTTGG | GGCCTTGACCTAGAAAT |
| rs701226 | ACGTTGGATGTGAAAGGTTGTCACTCACTG | ACGTTGGATGGGCAGTGATTCCCATTCTTC | CTCACTGAACCCCTAATG |
| rs6960872 | ACGTTGGATGGGTTTCTCAAATGCCAGGTC | ACGTTGGATGTCCCCTTGTTTGTGCCTAGC | TGCCAGGTCAGCTATATC |
| rs13219486 | ACGTTGGATGCATCACGAATCATTGGAGGC | ACGTTGGATGTAGATCAGCCAGAACAGTTG | CTCTCCCATGGTACTCATT |
| rs9314317 | ACGTTGGATGATACTGTGTCTTGGCAGCTC | ACGTTGGATGCCAAACTACCATTCACCGTC | GGATTTGCCCAGAATGTTA |
| rs2854108 | ACGTTGGATGTGGGTAACAGAGTGAGATCC | ACGTTGGATGGTTCAAATCTTAGCTCAGCC | AAGAGTGAGATCCTGTTAC |
| rs11102286 | ACGTTGGATGCCAAGTGACAGTGCTTACTC | ACGTTGGATGAGAGGCCATATCAGCTTTCC | CAGTGCTTACTCTACTCTTG |
| rs991697 | ACGTTGGATGGTACCTGTAATAGAGAAGGG | ACGTTGGATGGGAGAAAACCTATGGAAACG | CTCTTTCTTCAAGGTCGACA |
| rs11196030 | ACGTTGGATGGGACTGGAAACTTGATGTGG | ACGTTGGATGTATGCTGCCCAAAGTGCTTG | GATTGGGCCAAATGTGGTTT |
| rs7796370 | ACGTTGGATGTGTCCTTTTCTTCTACTCTC | ACGTTGGATGGACACCTAGCAGTAATATTG | GTTTCTTCTACTCTCTCCTTC |
| rs6743983 | ACGTTGGATGCCAGGTTCTGGACTATGTGC | ACGTTGGATGAATCTACAAACAAATAAGAG | GGCTGTCCACATTTGAATAAC |
| rs17630223 | ACGTTGGATGAGCTAGTTTCAGTGGAGTGC | ACGTTGGATGGGTCTCAAAAGGGATTGAGG | TTGCTACTTCCTTATTAACTCA |
| rs1875200 | ACGTTGGATGCTGGGACAGCTAACATAAGG | ACGTTGGATGTAGCCAATATGCCTTCCTGC | AAGGTAGACAAACTTTTGCATA |
| rs1887054 | ACGTTGGATGCCACTTCTAGATCTCTCCTG | ACGTTGGATGACCAATGGCCTCCCATAAAC | GGAGAGATCTCTCCTGGGAAAT |
| rs6429366 | ACGTTGGATGGCAGAGGAAATGGTTTCTGA | ACGTTGGATGCGAGTAGAGGAAAGAATGATG | GGACATTACTTCATCCTTTTACT |
| rs7707327 | ACGTTGGATGGGCTATGAAACTAAGTAAGAG | ACGTTGGATGTGAGTGTTATCCAAATGAG | CTATATTTGCACTTGCTACAATA |
| rs7948646 | ACGTTGGATGTAGGCCCTCACTGAAGTTAC | ACGTTGGATGCAATGGAAAGGAGAGCATGG | GGACATTAAGCCTAATTTAGCCTA |
| rs17767647 | ACGTTGGATGGACTGTGGAGTTGCTGAAAG | ACGTTGGATGCCAAGATTTCTCAGTATGTG | AAAGATAGCATGTTCTAGTGTTTA |
| rs1560073 | ACGTTGGATGTGGGTACATTTCGTTCACAC | ACGTTGGATGAATTTCTCTACCCTACCCCC | TTTAGTTCACACTTTCTCTTCTTCA |
| rs2826880 | ACGTTGGATGTTCTACACAGATATCAGCAC | ACGTTGGATGCAGTGTTAGTTCAAAAGGCTC | TCAGCACATATGAATCAAATGCATA |
| rs11658522 | ACGTTGGATGCTTTTCTGTGCTTTCCAAGG | ACGTTGGATGCAAGAGCGCTTAGAAAAATGC | GGGAGGTGCTTTCCAAGGTTTTTGC |
| rs1027902 | ACGTTGGATGACGCTGACGTGGGTAAAATG | ACGTTGGATGTCCAGGTAAGAAGTTGTCCC | CCACATGAAATTGCTTTTCTTACCTA |
| rs1469600 | ACGTTGGATGCTACCGTGTAGATATTCATC | ACGTTGGATGGCTTCAAAAAGAGTCCTGTC | CTACCGTGTAGATATTCATCAATAAT |
| rs9858318 | ACGTTGGATGGTGGGAGTACATGTAAGAGC | ACGTTGGATGCTACTGAGCATTTATTCTCC | GGGTTATTATATCATCTAGTGGGTGA |
| rs290186 | ACGTTGGATGTGGGAGACTATAAGCCTAAC | ACGTTGGATGTAAGTGCAGTGAGATGTGCC | CCTCGACTATAAGCCTAACTATCTACA |
| rs4465006 | ACGTTGGATGATACACACATACCACGTGGC | ACGTTGGATGCTATATCAAGAGAGCATGAAG | GGACATATTTAACTGTGTAAGTCTTGC |
| rs6481297 | ACGTTGGATGTAGAGCAAGGGTTGTCAAAC | ACGTTGGATGCCATTCTTAACACGCAGCTT | CCCCCTAAGAGACAAATCTAGCATATCA |
| rs3932338 | ACGTTGGATGAGATGGCAGACCTTGACTTG | ACGTTGGATGGCCAATCAAGGTTTCAAGAG | GGGGCTTGCAGAGATTATGACAGTCCAT |
| rs9965625 | ACGTTGGATGGGTAATGGCCATAGAGTAAA | ACGTTGGATGCAAAATGTTGGGATTACAGGC | GGTAATGAATCAGGTACAAAAATTAGAC |
| rs1886569 | ACGTTGGATGGAGACACAACACCCAAAATG | ACGTTGGATGTGGCTTAAGTGGAGTCTGTC | ACCCAAAATGTGAGGT |
| rs13172358 | ACGTTGGATGATGAGAGCCCCTCCTTTATG | ACGTTGGATGAGAATCAAAATGGAGTCAC | CCCGACTCCTATTTGTC |
| rs10791126 | ACGTTGGATGCTGGATGATCCATGAGAGAC | ACGTTGGATGGTCAGGCTCTGTGCTAAATG | GAGACAGGATCCATGTAA |
| rs4839595 | ACGTTGGATGTGTGGAAACCTGGCAACTTC | ACGTTGGATGGAGGCCAGCCAATTGTAAAC | GGCAACTTCATTAACATCT |
| rs10799384 | ACGTTGGATGCAGCCACAGACTGCATATAC | ACGTTGGATGGGGTCAAGATCATCAAGACG | GCATATACAACAATGGTCC |
| rs6569843 | ACGTTGGATGCAGGGCATTCTCAATAGAGT | ACGTTGGATGTTCCTCCTTGCATCCTCTTC | GGTGCAAAGACATGGAAGT |
| rs7148202 | ACGTTGGATGCTCTGATGGTAGTTTCTTTTG | ACGTTGGATGCCCCGAGAGGATATATTTTC | GTAGATTTGCTATCCATGTG |
| rs17225585 | ACGTTGGATGTGCATTATGGGATTGGCTTC | ACGTTGGATGCCAAGGGCAAAGGATAACTG | CCGTCCTTACATTGTCAAGTT |
| rs462509 | ACGTTGGATGTGGAGAGGGAAACAAGAGTG | ACGTTGGATGGCATGCTGTAAGGATGGTTG | GTAGGTTTTATTCTCAGTCTG |
| rs7653718 | ACGTTGGATGCATTCATTTTGTAATGAAAG | ACGTTGGATGTGGTAAAGAAGAAAATGCG | CGAATGTATTTCAACTCTTGTG |
| rs1403629 | ACGTTGGATGTTTGGGAAGCAGTACAGTAG | ACGTTGGATGCCTATCTCGAAGGTGTCAAC | GGAAGCAGTACAGTAGTATAAT |
| rs6599001 | ACGTTGGATGCCCAGGAAACATTGGCAAAG | ACGTTGGATGAGTGATAGAGCCTGTATTAC | CCCCTCCTAGAGTCAACTGTCTC |
| rs10516175 | ACGTTGGATGCTCTTTCCACTTCCACCTTC | ACGTTGGATGAGTTTCTCTTGGCAATGCCC | ATCTTGAGATCTTACTTTCCTTT |
| rs1027949 | ACGTTGGATGCAGTTTACTTACCCAGATCC | ACGTTGGATGTAGAGCTTCATTAGCTGTCC | CTGCTTGCCAAGTATGAAATTAT |
| rs8028364 | ACGTTGGATGGCTAAGGAAATGTCTGGAAG | ACGTTGGATGGACTAATGCCATGTAATGAG | CTCCGGAAGAGCTCTTTGTAAACT |
| rs2187132 | ACGTTGGATGCATTCCATTATATGAAATGGC | ACGTTGGATGTTCTTCCAGGGCTAAGGAAC | ACATTATATGAAATGGCAAAGTAG |
| rs2092107 | ACGTTGGATGGTGTTGAGCTGTGATACGTG | ACGTTGGATGGTCCTTCCAAAGACACCATC | CCCCCATTCATTGTAGGCTTGTCTC |
| rs2647571 | ACGTTGGATGTGCCCCTATCACCTATTCTC | ACGTTGGATGAAAGTCCAATCACCCGTGAG | TTTCCATTTAGGCATTTAGGTAAAC |
| rs370176 | ACGTTGGATGACTATTGCAGGGTAGAATGG | ACGTTGGATGGTGTTAACAAGGAACCACTG | GTGCAAGAGCATGAGTTGTCTTATT |
| rs270707 | ACGTTGGATGCCCAGCACCAATCTCCTTTC | ACGTTGGATGGAGAAATTAGACTGCGTGCC | TCTCTTTTTAATGTGTTTTAAGGTAT |
| rs2155411 | ACGTTGGATGCTCTGAGCCTCAATGTTTCC | ACGTTGGATGGGAATTTCTCATACTCTGTC | GAGGAAGCCATCTTATAGGATTGCTG |
| rs7946409 | ACGTTGGATGCCTCTTGATATCTTCTATTG | ACGTTGGATGACTATCCCTTAGAAAGAAC | CTCTTGATATCTTCTATTGAATTTAAC |
| rs9881109 | ACGTTGGATGAGTCTACAGGGTGGTATTAC | ACGTTGGATGGCAACAAACAGATATTCAGTG | TATGGTGGTATTACTAGTATTTTGTAT |
| rs11154775 | ACGTTGGATGCCTCAGAAAAGATGAGTCAAG | ACGTTGGATGAGGCCACCCTCAGAAAATAG | AGTTTCAAAATTTTATTTCATTTATTGA |
| rs283589 | ACGTTGGATGGGAGAATATAAACTTCCTA | ACGTTGGATGTCACTTCTTCCTGGTGTGAA | AATATAATGGTGTAACAATAAATTCTAA |
| rs13421493 | ACGTTGGATGAGTTATTTATAAGCAAATCC | ACGTTGGATGGGAATTATTTCTGGGTGAAG | AAGCAAATCCCACTGA |
| rs7048859 | ACGTTGGATGTCACCATGTTGCCTAGGCTG | ACGTTGGATGCAGGATTAAAAGTCCAGCTC | AGGCTGGTCTTGAACTC |
| rs10873824 | ACGTTGGATGCCTACCTTATTAGGATTTGGG | ACGTTGGATGCACTTTAAAAGGGTGAAATG | TGTTCATAACAGCTTCTTC |
| rs3111458 | ACGTTGGATGCACTTCCCCAGAGATAAACC | ACGTTGGATGCGTTTGCTCAAAACCTATCC | TGGTTTGTGTTTCCAGATA |
| rs157861 | ACGTTGGATGAGTGAGAGGATGGAGAACTG | ACGTTGGATGTGGAGGTAGAAAGGGACAAC | GATGGAGAACTGGTAAAGA |
| rs9911870 | ACGTTGGATGAGTCTGGGAAATTTGGGCTC | ACGTTGGATGACTCACCCTACTCAAAAGCC | TACTGACCGTTTACCAAATA |
| rs17333350 | ACGTTGGATGATATCAAGTGGGAGGTAGTC | ACGTTGGATGCACATTGACACTGATATCAC | CGGTAGTCTCTACAACTGTC |
| rs17595877 | ACGTTGGATGGGGCTTAGAACCTGAAGATG | ACGTTGGATGATGGGAACAATAGGTATAGG | ATTCTACTAAAGGCTTTTGAA |
| rs11766009 | ACGTTGGATGCCGGGAAATACCCAGTCATT | ACGTTGGATGTTCCGTCCTTTTCAGGACTC | GCTCCAAATTACTGCCTAGAGAG |
| rs472826 | ACGTTGGATGCATCTGATGTTTGGCACCAC | ACGTTGGATGGAGCTTTCCAGACAAGGATG | ACCACTTAGCAAGATTCCATTAATT |
| rs1522012 | ACGTTGGATGATGTAGACACAGGCTGAACC | ACGTTGGATGGACATTCAACATCTTTTGAG | TACTCAAAGAATATATCAATTGCCA |
| rs10943471 | ACGTTGGATGTTTTGAAAATTGATAGCTTG | ACGTTGGATGTTCTGACACTTTTCAGATG | TGATAGCTTGTTTATAAAGAATTTT |
| rs7990079 | ACGTTGGATGGGGTGAATTCCTTTTTTGTG | ACGTTGGATGGTAGGAAAAAGCTTTAACCTC | GGTTTTCTAGGATAACAATAAATGGG |
| rs7305157 | ACGTTGGATGAAGCCATTTCTGTACAGTTG | ACGTTGGATGGAGCAGTGGTTCAGTATTTG | CTCACAGTAGTTAAATTTTATAAAGTC |
