# Supplementary material for: Genetic Variants Underlying Risk of Intracranial Aneurysms: Insights from a GWAS in Portugal
Source: PLoS One. 2015 Jul 17;10(7):e0133422. doi: 10.1371/journal.pone.0133422 (PMC4505843; doi:10.1371/journal.pone.0133422)
Supplement: S2 Table — (DOCX) [file pone.0133422.s004.docx]

**S2 Table. 113 single nucleotide polymorphisms (SNPs) with |RAS_diff_|≥13.0% in the pool-based genome-wide association study for intracranial aneurysms**

| **SNP** | **Affymetrix ID** | **Chr.** | **Position (bp)** | **Gene (nearest gene)** | **Alleles**  **1:2** | **AF** | **\|RAS_diff_\|** |
| --- | --- | --- | --- | --- | --- | --- | --- |
| rs6592222 | SNP_A-1908146 | 11 | 84661830 | *DLG2* | A:G | 0.367 | 17.5% |
| rs1560073 | SNP_A-2265231 | 5 | 5713635 | *ICE1* (223 kb) | C:T | 0.770 | 17.2% |
| rs991697 | SNP_A-4214675 | 11 | 7376305 | *SYT9* | A:G | 0.323 | 16.2% |
| rs7796370 | SNP_A-1933501 | 7 | 91901716 | *ANKIB1* | A:C | 0.217 | 15.7% |
| rs472826 | SNP_A-8316482 | 11 | 107623767 | *SLC35F2* (38 kb) | G:T | 0.350 | 15.4% |
| rs1005318 | SNP_A-1870840 | 17 | 69008873 | *CASC17* (85 kb) | A:G | 0.226 | 15.3% |
| rs11658522 | SNP_A-8351864 | 17 | 59974651 | *INTS2* | A:G | 0.181 | 15.2% |
| rs17595877 | SNP_A-2083285 | 9 | 6933222 | *KDM4C* | C:T | 0.341 | 15.2% |
| rs9881109 | SNP_A-8345946 | 3 | 179430156 | *USP13* | A:G | 0.217 | 15.1% |
| rs17225585 | SNP_A-1830202 | 17 | 69370430 | *CASC17* (172 kb) | G:T | 0.123 | 15.1% |
| rs270707 | SNP_A-1954993 | 1 | 45049458 | *RNF220* | A:G | 0.478 | 15.1% |
| rs13219486 | SNP_A-8332709 | 6 | 15801167 | *DTNBP1* (138 kb) | C:T | 0.385 | 15.0% |
| rs2647571 | SNP_A-8462518 | 11 | 5444793 | *HBG2* | G:T | 0.358 | 15.0% |
| rs1875200 | SNP_A-8686738 | 5 | 72426988 | *TMEM171* | A:G | 0.332 | 15.0% |
| rs17053831 | SNP_A-8659937 | 5 | 156145239 | *SGCD* | A:G | 0.062 | 14.9% |
| rs7463038 | SNP_A-2035616 | 8 | 38587043 | *TACC1* | A:G | 0.125 | 14.9% |
| rs6743983 | SNP_A-2166008 | 2 | 53826778 | *GPR75-ASB3* | A:C | 0.460 | 14.9% |
| rs13135261 | SNP_A-1976659 | 4 | 30773963 | *PCDH7* | A:G | 0.274 | 14.9% |
| rs7048859 | SNP_A-8356935 | 9 | 126156503 | *DENND1A* | C:T | 0.142 | 14.9% |
| rs10006104 | SNP_A-8391851 | 4 | 86841160 | *ARHGAP24* | C:T | 0.765 | 14.9% |
| rs1027949 | SNP_A-4244455 | 12 | 110430968 | *GIT2* | C:T | 0.168 | 14.7% |
| rs6599001 | SNP_A-1850672 | 3 | 39074151 | *WDR48* (19 kb) | C:T | 0.119 | 14.7% |
| rs10516175 | SNP_A-1975880 | 4 | 5671477 | *EVC2* | A:G | 0.115 | 14.6% |
| rs283589 | SNP_A-8488228 | 6 | 51016787 | *TFAP2B* (202 kb) | A:G | 0.296 | 14.6% |
| rs3111458 | SNP_A-2214924 | 7 | 112187966 | *LSMEM1* (57 kb) | G:T | 0.327 | 14.6% |
| rs668001 | SNP_A-1924426 | 13 | 26005056 | *ATP8A2* | C:T | 0.261 | 14.6% |
| rs12991586 | SNP_A-1952871 | 2 | 118872396 | *INSIG2* (5 kb) | A:G | 0.027 | 14.6% |
| rs1887054 | SNP_A-2140662 | 1 | 92099741 | *HSP90B3P* | A:G | 0.296 | 14.6% |
| rs8028364 | SNP_A-8699072 | 15 | 81483730 | *IL16* | C:G | 0.223 | 14.5% |
| rs1403629 | SNP_A-8290110 | 10 | 75773362 | *VCL* | C:G | 0.250 | 14.4% |
| rs10791126 | SNP_A-4297537 | 11 | 131032248 | *NTM* (208 kb) | A:T | 0.381 | 14.4% |
| rs2854108 | SNP_A-2121853 | 1 | 19973920 | *NBL1* | A:G | 0.204 | 14.4% |
| rs9314317 | SNP_A-4212871 | 8 | 25833952 | *EBF2* | A:G | 0.155 | 14.4% |
| rs9965625 | SNP_A-2178958 | 18 | 52830279 | *TCF4* (59 kb) | A:G | 0.451 | 14.3% |
| rs2826880 | SNP_A-8342277 | 21 | 22904654 | *NCAM2* | A:G | 0.486 | 14.3% |
| rs1027902 | SNP_A-1966715 | 2 | 211287365 | *LANCL1* (8 kb) | C:T | 0.482 | 14.2% |
| rs13355489 | SNP_A-8501508 | 5 | 156664822 | *ITK* | C:T | 0.097 | 14.2% |
| rs12691433 | SNP_A-8569891 | 7 | 154989404 | *INSIG1* (100 kb) | C:T | 0.243 | 14.2% |
| rs652264 | SNP_A-8326520 | 18 | 3589668 | *DLGAP1* | A:G | 0.438 | 14.1% |
| rs10915933 | SNP_A-8650536 | 1 | 226279050 | *H3F3A* (196 kb) | A:G | 0.350 | 14.1% |
| rs7653718 | SNP_A-1852692 | 3 | 29021245 | *RBMS3* (301 kb) | C:T | 0.434 | 14.1% |
| rs9911870 | SNP_A-2272147 | 17 | 3212858 | *OR3A4P* | A:G | 0.119 | 14.1% |
| rs1522012 | SNP_A-2168785 | 13 | 87618065 | *SLITRK5* (707 kb) | A:G | 0.228 | 14.0% |
| rs6481297 | SNP_A-2166324 | 10 | 59333310 | *IPMK* (617 kb) | C:T | 0.438 | 14.0% |
| rs2949574 | SNP_A-2270348 | 15 | 31251781 | *MTMR10* | C:T | 0.420 | 13.9% |
| rs542892 | SNP_A-4215265 | 11 | 84791038 | *DLG2* | C:T | 0.388 | 13.9% |
| rs11102286 | SNP_A-8292920 | 1 | 112024624 | *ADORA3* | A:G | 0.274 | 13.9% |
| rs2274758 | SNP_A-2175901 | 13 | 75894015 | *TBC1D4* | C:T | 0.456 | 13.9% |
| rs701226 | SNP_A-1988699 | 1 | 233767059 | *KCNK1* | C:T | 0.230 | 13.9% |
| rs1357938 | SNP_A-8430713 | 7 | 144729189 | *TPK1* (196 kb) | A:G | 0.171 | 13.9% |
| rs26118 | SNP_A-2082552 | 5 | 14173943 | *TRIO* | A:G | 0.062 | 13.8% |
| rs1922648 | SNP_A-1935562 | 4 | 160458424 | *RAPGEF2* (177 kb) | A:G | 0.044 | 13.8% |
| rs13172358 | SNP_A-8589506 | 5 | 141484125 | *NDFIP1* (4 kb) | C:G | 0.137 | 13.8% |
| rs1744895 | SNP_A-8544226 | 6 | 21680899 | *LINC00340* | C:G | 0.400 | 13.8% |
| rs7707327 | SNP_A-8430468 | 5 | 5723376 | *ICE1* (232 kb) | A:G | 0.204 | 13.8% |
| rs11154775 | SNP_A-8499009 | 6 | 134847247 | *ALDH8A1* | C:G | 0.420 | 13.8% |
| rs11766009 | SNP_A-8563170 | 7 | 97177702 | *TAC1* (183 kb) | C:T | 0.181 | 13.7% |
| rs270706 | SNP_A-8595070 | 1 | 45048818 | *RNF220* | C:G | 0.460 | 13.7% |
| rs7224497 | SNP_A-1833664 | 17 | 3212779 | *OR3A4P* | C:T | 0.119 | 13.7% |
| rs4667622 | SNP_A-4232316 | 2 | 171004185 | *MYO3B* (30 kb) | A:G | 0.487 | 13.7% |
| rs9857513 | SNP_A-2176102 | 3 | 161598035 | *OTOL1* (377 kb) | C:T | 0.252 | 13.7% |
| rs11756174 | SNP_A-8404877 | 6 | 6198639 | *F13A1* | A:G | 0.168 | 13.7% |
| rs17333350 | SNP_A-2081994 | 5 | 101814609 | *SLCO6A1* | A:G | 0.288 | 13.7% |
| rs589658 | SNP_A-8439395 | 11 | 78702526 | *TENM4* | A:G | 0.473 | 13.6% |
| rs3934723 | SNP_A-1985801 | 1 | 221980064 | *DUSP10* (65 kb) | C:G | 0.335 | 13.6% |
| rs462509 | SNP_A-8607613 | 4 | 187602279 | *FAT1* | C:T | 0.150 | 13.6% |
| rs157861 | SNP_A-2313133 | 1 | 165378161 | *RXRG* | C:G | 0.221 | 13.6% |
| rs7157956 | SNP_A-4234316 | 14 | 70415245 | *SMOC1* | C:T | 0.482 | 13.6% |
| rs9500325 | SNP_A-8697340 | 6 | 58547058 | *GUSBP4* (260 kb) | C:T | 0.208 | 13.6% |
| rs2155411 | SNP_A-1879391 | 11 | 84659922 | *DLG2* | C:T | 0.366 | 13.5% |
| rs1886569 | SNP_A-8418944 | 6 | 51038157 | *TFAP2B* (223 kb) | C:T | 0.305 | 13.5% |
| rs11072994 | SNP_A-1926436 | 15 | 81519313 | *IL16* | A:T | 0.223 | 13.5% |
| rs9858318 | SNP_A-2137583 | 3 | 158223291 | *RSRC1* | A:G | 0.496 | 13.5% |
| rs10732827 | SNP_A-1914818 | 10 | 85891907 | *GHITM* (7 kb) | A:G | 0.367 | 13.5% |
| rs10014056 | SNP_A-8518053 | 4 | 190909432 | *TUBB4Q* (4 kb) | A:G | 0.296 | 13.4% |
| rs370176 | SNP_A-2240197 | 5 | 101595558 | *SLCO4C1* | A:G | 0.243 | 13.4% |
| rs2837636 | SNP_A-8338864 | 21 | 41826974 | *DSCAM* | C:T | 0.336 | 13.4% |
| rs7946409 | SNP_A-1933869 | 11 | 38573340 | *LRRC4C* (1562 kb) | C:G | 0.319 | 13.4% |
| rs4918006 | SNP_A-4251646 | 10 | 95768811 | *PLCE1* | A:G | 0.263 | 13.4% |
| rs16977458 | SNP_A-8515820 | 15 | 57686011 | *CGNL1* | G:T | 0.320 | 13.4% |
| rs4465006 | SNP_A-8286209 | 8 | 65525439 | *CYP7B1* | C:T | 0.326 | 13.3% |
| rs1499901 | SNP_A-4242243 | 3 | 112905278 | *BOC* (25 kb) | A:G | 0.323 | 13.3% |
| rs1469600 | SNP_A-8561221 | 13 | 75895847 | *TBC1D4* | G:T | 0.450 | 13.3% |
| rs10457678 | SNP_A-2263771 | 6 | 139122240 | *ECT2L* | A:G | 0.221 | 13.3% |
| rs10834648 | SNP_A-8641707 | 11 | 3645345 | *TRPC2* (2 kb) | A:G | 0.257 | 13.3% |
| rs7865885 | SNP_A-8599168 | 9 | 100318739 | *TMOD1* | A:G | 0.496 | 13.2% |
| rs6864279 | SNP_A-2046643 | 5 | 178515060 | *ZNF354C* (8 kb) | G:T | 0.420 | 13.2% |
| rs2187132 | SNP_A-2083586 | 11 | 104500178 | *CASP12* (265 kb) | A:G | 0.226 | 13.2% |
| rs6782003 | SNP_A-4263574 | 3 | 67967713 | *SUCLG2-AS1* | A:C | 0.115 | 13.2% |
| rs10183045 | SNP_A-4228875 | 2 | 41877732 | *C2orf91* (285 kb) | G:T | 0.226 | 13.2% |
| rs4839595 | SNP_A-1846525 | 3 | 143163169 | *SLC9A9* | A:G | 0.478 | 13.2% |
| rs290186 | SNP_A-8472172 | 11 | 85395652 | *CCDC89* | C:T | 0.332 | 13.2% |
| rs7305157 | SNP_A-8487682 | 12 | 24920581 | *BCAT1* (42 kb) | A:C | 0.111 | 13.2% |
| rs7148202 | SNP_A-1958882 | 14 | 74452465 | *ENTPD5* | C:T | 0.394 | 13.2% |
| rs1866970 | SNP_A-1866043 | 1 | 30103672 | *PTPRU* (450 kb) | A:G | 0.314 | 13.2% |
| rs2092107 | SNP_A-8447892 | 6 | 16203561 | *GMPR* (35 kb) | G:T | 0.115 | 13.2% |
| rs10799384 | SNP_A-8399760 | 1 | 227297555 | *CDC42BPA* | A:G | 0.152 | 13.2% |
| rs209573 | SNP_A-2006219 | 1 | 40838636 | *SMAP2* | C:G | 0.378 | 13.2% |
| rs932797 | SNP_A-8416460 | 6 | 41562309 | *FOXP4* | C:T | 0.181 | 13.1% |
| rs7990079 | SNP_A-8696316 | 13 | 40045976 | *LHFP* | G:T | 0.371 | 13.1% |
| rs17767647 | SNP_A-8602067 | 18 | 38681767 | *KC6* (378 kb) | C:T | 0.208 | 13.1% |
| rs17516032 | SNP_A-2209904 | 15 | 91686453 | *SV2B* | A:G | 0.389 | 13.1% |
| rs17630223 | SNP_A-2252593 | 3 | 158379172 | *GFM1* | C:G | 0.181 | 13.1% |
| rs10416963 | SNP_A-1848692 | 19 | 17762890 | *UNC13A* | A:G | 0.385 | 13.1% |
| rs7948646 | SNP_A-2012865 | 11 | 84785991 | *DLG2* | A:C | 0.239 | 13.1% |
| rs10873824 | SNP_A-8397975 | 1 | 87688394 | *LMO4* (106 kb) | A:G | 0.300 | 13.0% |
| rs3932338 | SNP_A-1827607 | 5 | 23742976 | *PRDM9* (214 kb) | A:G | 0.438 | 13.0% |
| rs11196030 | SNP_A-2032128 | 10 | 114401543 | *VTI1A* | C:G | 0.390 | 13.0% |
| rs6960872 | SNP_A-2145873 | 7 | 144702641 | *TPK1* (170 kb) | A:C | 0.491 | 13.0% |
| rs6569843 | SNP_A-8670883 | 6 | 133061091 | *VNN2* (4 kb) | C:T | 0.288 | 13.0% |
| rs6429366 | SNP_A-8362666 | 1 | 242767005 | *PLD5* (79 kb) | C:T | 0.460 | 13.0% |
| rs13421493 | SNP_A-8367945 | 2 | 220844439 | *SLC4A3* (338 kb) | A:T | 0.363 | 13.0% |
| rs10943471 | SNP_A-4211764 | 6 | 78269962 | *HTR1B* (96 kb) | A:G | 0.270 | 13.0% |

The polymorphisms are listed in decreasing order of |RAS_diff_|. When a SNP is not located in a gene, the name and distance to the nearest RefSeq gene is indicated.

Chr.: Chromosome; bp: Base pair position (Affymetrix GenomeWideSNP_6 Annotations, release 32); kb: Kilobase pairs; AF: Allele frequency of allele 2 in the CEU HapMap dataset (release 28, PhaseII+PhaseIII, August 2010); |RAS_diff_|: Absolute value of the relative allele score difference.
