# Supplementary material for: Genetic Variants Underlying Risk of Intracranial Aneurysms: Insights from a GWAS in Portugal
Source: PLoS One. 2015 Jul 17;10(7):e0133422. doi: 10.1371/journal.pone.0133422 (PMC4505843; doi:10.1371/journal.pone.0133422)
Supplement: S3 Table — (DOCX) [file pone.0133422.s005.docx]

**S3 Table. Association results of the single nucleotide polymorphisms (SNPs) tested in the technical validation and independent replication phases**

| **SNP** | **Chr.** | **Gene/nearest gene (kb)** | **Allele** | **Discovery dataset** | | | | **Replication dataset** | | | |
| --- | --- | --- | --- | --- | --- | --- | --- | --- | --- | --- | --- |
|  |  |  |  | **F_cases_** | **F_controls_** | ***P*** | **OR[95% CI]** | **F_cases_** | **F_controls_** | ***P*** | **OR[95% CI]** |
| rs2854108 | 1 | *NBL1* | G | 0.338 | 0.198 | **2.07E-03** | **2.07[1.30-3.31]** | 0.179 | 0.268 | **1.84E-02** | **0.63[0.42-0.93]** |
| rs1866970 | 1 | *PTPRU* (450 kb) | T | 0.354 | 0.189 | **3.43E-04** | **2.35[1.46-3.77]** | 0.278 | 0.232 | 1.94E-01 | - |
| rs209573 | 1 | *SMAP2* | C | 0.606 | 0.445 | **1.68E-03** | **1.92[1.28-2.89]** | 0.582 | 0.559 | 5.94E-01 | - |
| rs270706 | 1 | *RNF220* | G | 0.596 | 0.400 | **1.41E-04** | **2.21[1.47-3.34]** | 0.516 | 0.486 | 4.62E-01 | - |
| rs270707 | 1 | *RNF220* | A | 0.586 | 0.390 | **1.37E-04** | **2.21[1.47-3.33]** | 0.515 | 0.472 | 2.83E-01 | - |
| rs10873824 | 1 | *LMO4* (106 kb) | A | 0.293 | 0.154 | **1.21E-03** | **2.28[1.37-3.78]** | 0.250 | 0.251 | 8.84E-01 | - |
| rs1887054 | 1 | *HSP90B3P* | A | 0.768 | 0.621 | **1.86E-03** | **2.02[1.29-3.15]** | 0.684 | 0.687 | 9.55E-01 | - |
| rs11102286 | 1 | *ADORA3* | G | 0.278 | 0.121 | **1.44E-04** | **2.80[1.62-4.82]** | 0.304 | 0.246 | 1.16E-01 | - |
| rs157861 | 1 | *RXRG* | C | 0.869 | 0.731 | **7.40E-04** | **2.44[1.44-4.13]** | 0.821 | 0.792 | 3.30E-01 | - |
| rs3934723 | 1 | *DUSP10* (65 kb) | C | 0.776 | 0.599 | **2.06E-04** | **2.31[1.48-3.62]** | 0.598 | 0.655 | 1.32E-01 | - |
| rs10915933 | 1 | *H3F3A* (196 kb) | A | 0.475 | 0.258 | **1.28E-05** | **2.60[1.68-4.01]** | 0.321 | 0.405 | **2.25E-02** | **0.68[0.49-0.95]** |
| rs10799384 | 1 | *CDC42BPA* | A | 0.288 | 0.137 | **3.66E-04** | **2.54[1.51-4.28]** | 0.163 | 0.228 | **4.12E-02** | **0.65[0.43-0.99]** |
| rs701226 | 1 | *KCNK1* | C | 0.354 | 0.181 | **1.61E-04** | **2.47[1.53-3.98]** | 0.235 | 0.243 | 7.78E-01 | - |
| rs6429366 | 1 | *PLD5* (79 kb) | C | 0.596 | 0.412 | **3.41E-04** | **2.10[1.40-3.17]** | 0.500 | 0.518 | 6.60E-01 | - |
| rs10183045 | 2 | *C2orf91* (285 kb) | T | 0.874 | 0.764 | **5.20E-03** | **2.14[1.25-3.68]** | 0.837 | 0.828 | 7.14E-01 | - |
| rs6743983 | 2 | *GPR75-ASB3* | A | 0.586 | 0.396 | **2.11E-04** | **2.16[1.43-3.26]** | 0.541 | 0.516 | 5.37E-01 | - |
| rs4667622 | 2 | *MYO3B* (30 kb) | G | 0.593 | 0.418 | **6.84E-04** | **2.03[1.35-3.06]** | 0.592 | 0.482 | **1.03E-02** | **1.55[1.11-2.17]** |
| rs1027902 | 2 | *LANCL1* (8 kb) | T | 0.665 | 0.494 | **8.84E-04** | **2.03[1.33-3.09]** | 0.619 | 0.624 | 9.81E-01 | - |
| rs13421493 | 2 | *SLC4A3* (338 kb) | T | 0.763 | 0.582 | **1.76E-04** | **2.30[1.48-3.58]** | 0.689 | 0.654 | 3.25E-01 | - |
| rs7653718 | 3 | *RBMS3* (301 kb) | T | 0.684 | 0.529 | **2.28E-03** | **1.93[1.26-2.94]** | 0.621 | 0.600 | 5.14E-01 | - |
| rs6599001 | 3 | *WDR48* (19 kb) | C | 0.197 | 0.099 | **7.48E-03** | **2.23[1.23-4.07]** | 0.189 | 0.114 | **7.90E-03** | **1.75[1.15-2.66]** |
| rs6782003 | 3 | *SUCLG2-AS1* | A | 0.924 | 0.791 | **1.84E-04** | **3.22[1.70-6.08]** | 0.883 | 0.868 | 5.70E-01 | - |
| rs1499901 | 3 | *BOC* (25 kb) | T | 0.760 | 0.600 | **8.49E-04** | **2.11[1.36-3.29]** | 0.670 | 0.712 | 2.42E-01 | - |
| rs4839595 | 3 | *SLC9A9* | G | 0.672 | 0.489 | **3.06E-04** | **2.14[1.41-3.24]** | 0.510 | 0.514 | 9.96E-01 | - |
| rs9858318 | 3 | *RSRC1* | A | 0.599 | 0.443 | **3.15E-03** | **1.88[1.23-2.87]** | 0.581 | 0.545 | 3.27E-01 | - |
| rs17630223 | 3 | *GFM1* | G | 0.859 | 0.789 | 7.46E-02 | - | - | - | - | - |
| rs9857513 | 3 | *OTOL1* (377 kb) | C | 0.389 | 0.242 | **2.10E-03** | **2.00[1.28-3.11]** | 0.357 | 0.305 | 1.15E-01 | - |
| rs9881109 | 3 | *USP13* | G | 0.200 | 0.090 | **2.85E-03** | **3.49[1.72-7.08]** | 0.134 | 0.119 | 6.22E-01 | - |
| rs10516175 | 4 | *EVC2* | A | 0.187 | 0.062 | **2.84E-04** | **2.04[1.31-3.16]** | 0.175 | 0.125 | 7.73E-02 | - |
| rs13135261 | 4 | *PCDH7* | A | 0.753 | 0.599 | **1.35E-03** | **2.09[1.28-3.40]** | 0.638 | 0.673 | 3.69E-01 | - |
| rs10006104 | 4 | *ARHGAP24* | T | 0.828 | 0.698 | **1.54E-04** | **2.11[1.36-3.29]** | 0.821 | 0.784 | 2.21E-01 | - |
| rs462509 | 4 | *FAT1* | T | 0.854 | 0.769 | **3.52E-02** | **1.75[1.04-2.95]** | 0.837 | 0.827 | 6.66E-01 | - |
| rs1560073 | 5 | *ICE1* (223 kb) | C | 0.343 | 0.150 | **1.49E-05** | **2.96[1.79-4.90]** | 0.330 | 0.261 | 3.72E-01 | - |
| rs7707327 | 5 | *ICE1* (232 kb) | G | 0.301 | 0.115 | **1.01E-05** | **3.30[1.91-5.71]** | 0.199 | 0.225 | 4.31E-01 | - |
| rs3932338 | 5 | *PRDM9* (214 kb) | A | 0.667 | 0.549 | **1.92E-02** | **1.64[1.08-2.48]** | 0.758 | 0.617 | **2.67E-04** | **1.93[1.35-2.77]** |
| rs1875200 | 5 | *TMEM171* | G | 0.505 | 0.286 | **1.30E-05** | **2.55[1.67-3.90]** | 0.449 | 0.381 | 7.12E-02 | - |
| rs370176 | 5 | *SLCO4C1* | T | 0.823 | 0.725 | **2.21E-02** | **1.76[1.08-2.88]** | 0.776 | 0.796 | 5.82E-01 | - |
| rs17333350 | 5 | *SLCO6A1* | A | 0.753 | 0.599 | **1.35E-03** | **2.04[1.31-3.16]** | 0.719 | 0.707 | 7.73E-01 | - |
| rs13172358 | 5 | *NDFIP1* (4 kb) | G | 0.157 | 0.044 | **3.02E-04** | **4.04[1.80-9.04]** | 0.087 | 0.087 | 9.42E-01 | - |
| rs6864279 | 5 | *ZNF354C* (8 kb) | T | 0.364 | 0.220 | **2.12E-03** | **2.03[1.29-3.20]** | 0.330 | 0.263 | 3.65E-01 | - |
| rs11756174 | 6 | *F13A1* | A | 0.894 | 0.775 | **1.67E-03** | **2.45[1.39-4.34]** | 0.827 | 0.831 | 9.09E-01 | - |
| rs13219486 | 6 | *DTNBP1* (138 kb) | T | 0.646 | 0.478 | **9.35E-04** | **2.00[1.32-3.01]** | 0.551 | 0.554 | 9.48E-01 | - |
| rs2092107 | 6 | *GMPR* (35 kb) | A | 0.944 | 0.833 | **5.22E-04** | **3.40[1.65-7.01]** | 0.913 | 0.906 | 9.16E-01 | - |
| rs932797 | 6 | *FOXP4* | C | 0.152 | 0.055 | **2.18E-03** | **3.07[1.46-6.48]** | 0.102 | 0.119 | 4.91E-01 | - |
| rs283589 | 6 | *TFAP2B* (202 kb) | G | 0.320 | 0.134 | **2.64E-05** | **3.04[1.79-5.18]** | 0.202 | 0.228 | 5.27E-01 | - |
| rs1886569 | 6 | *TFAP2B* (223 kb) | G | 0.318 | 0.144 | **7.00E-05** | **2.76[1.66-4.61]** | 0.235 | 0.241 | 9.52E-01 | - |
| rs9500325 | 6 | *GUSBP4* (260 kb) | T | 0.866 | 0.731 | **1.04E-03** | **2.38[1.41-4.03]** | 0.830 | 0.788 | 1.69E-01 | - |
| rs10943471 | 6 | *HTR1B* (96 kb) | G | 0.247 | 0.121 | **1.56E-03** | **2.39[1.38-4.15]** | 0.250 | 0.178 | **2.73E-02** | **1.51[1.05-2.19]** |
| rs6569843 | 6 | *VNN2* (4 kb) | C | 0.540 | 0.335 | **5.71E-05** | **2.33[1.54-3.53]** | 0.495 | 0.432 | 1.08E-01 | - |
| rs11154775 | 6 | *ALDH8A1* | C | 0.702 | 0.566 | **5.86E-03** | **1.81[1.18-2.76]** | 0.628 | 0.629 | 9.66E-01 | - |
| rs10457678 | 6 | *ECT2L* | A | 0.732 | 0.604 | **8.01E-03** | **1.79[1.16-2.76]** | 0.704 | 0.734 | 2.99E-01 | - |
| rs7796370 | 7 | *ANKIB1* | C | 0.268 | 0.111 | **1.18E-04** | **2.92[1.67-5.13]** | 0.211 | 0.170 | 2.25E-01 | - |
| rs11766009 | 7 | *TAC1* (183 kb) | C | 0.187 | 0.077 | **1.68E-03** | **2.76[1.44-5.29]** | 0.117 | 0.120 | 8.72E-01 | - |
| rs3111458 | 7 | *LSMEM1* (57 kb) | G | 0.773 | 0.621 | **1.25E-03** | **2.08[1.33-3.25]** | 0.607 | 0.622 | 7.87E-01 | - |
| rs6960872 | 7 | *TPK1* (170 kb) | A | 0.692 | 0.495 | **8.86E-05** | **2.30[1.51-3.49]** | 0.592 | 0.543 | 2.12E-01 | - |
| rs12691433 | 7 | *INSIG1* (100 kb) | T | 0.343 | 0.214 | **5.17E-03** | **1.92[1.21-3.04]** | 0.189 | 0.273 | **2.84E-02** | **0.65[0.45-0.96]** |
| rs9314317 | 8 | *EBF2* | A | 0.874 | 0.750 | **2.73E-03** | **2.31[1.32-4.02]** | 0.879 | 0.842 | 3.58E-01 | - |
| rs7463038 | 8 | *TACC1* | G | 0.162 | 0.055 | **9.23E-04** | **3.32[1.58-6.96]** | 0.133 | 0.109 | 3.63E-01 | - |
| rs4465006 | 8 | *CYP7B1* | T | 0.686 | 0.528 | **1.86E-03** | **1.95[1.28-2.97]** | 0.547 | 0.639 | **2.15E-02** | **0.69[0.50-0.95]** |
| rs17595877 | 9 | *KDM4C* | T | 0.866 | 0.702 | **1.14E-04** | **2.74[1.62-4.62]** | 0.646 | 0.683 | 4.20E-01 | - |
| rs7865885 | 9 | *TMOD1* | A | 0.601 | 0.429 | **7.77E-04** | **2.01[1.33-3.02]** | 0.418 | 0.506 | **4.18E-02** | **0.72[0.53-0.99]** |
| rs7048859 | 9 | *DENND1A* | C | 0.939 | 0.844 | **2.73E-03** | **2.86[1.40-5.80]** | 0.862 | 0.913 | **3.62E-02** | **0.60[0.38-0.97]** |
| rs6481297 | 10 | *IPMK* (617 kb) | C | 0.641 | 0.440 | **7.91E-05** | **2.28[1.51-3.44]** | 0.628 | 0.596 | 4.56E-01 | - |
| rs1403629 | 10 | *VCL* | C | 0.333 | 0.220 | **1.37E-02** | **1.78[1.12-2.81]** | 0.245 | 0.273 | 4.02E-01 | - |
| rs10732827 | 10 | *GHITM* (7 kb) | G | 0.672 | 0.495 | **4.57E-04** | **2.09[1.38-3.17]** | 0.597 | 0.585 | 7.01E-01 | - |
| rs4918006 | 10 | *PLCE1* | G | 0.318 | 0.170 | **8.47E-04** | **2.27[1.39-3.71]** | 0.270 | 0.279 | 8.53E-01 | - |
| rs11196030 | 10 | *VTI1A* | C | 0.465 | 0.286 | **3.28E-04** | **2.17[1.42-3.32]** | 0.403 | 0.393 | 8.98E-01 | - |
| rs2647571 | 11 | *HBG2* | C | 0.424 | 0.280 | **3.38E-03** | **2.28[1.51-3.44]** | 0.352 | 0.397 | 2.26E-01 | - |
| rs991697 | 11 | *SYT9* | A | 0.763 | 0.593 | **4.04E-04** | **1.78[1.12-2.81]** | 0.684 | 0.652 | 3.23E-01 | - |
| rs7946409 | 11 | *LRRC4C* (1562 kb) | C | 0.399 | 0.275 | **1.15E-02** | **1.75[1.13-2.70]** | 0.335 | 0.346 | 8.35E-01 | - |
| rs589658 | 11 | *TENM4* | T | 0.621 | 0.495 | **1.29E-02** | **1.68[1.11-2.52]** | 0.520 | 0.514 | 9.96E-01 | - |
| rs2155411 | 11 | *DLG2* | C | 0.439 | 0.247 | **8.50E-05** | **2.39[1.54-3.70]** | 0.362 | 0.387 | 6.44E-01 | - |
| rs6592222 | 11 | *DLG2* | G | 0.434 | 0.247 | **1.27E-04** | **2.34[1.51-3.62]** | 0.347 | 0.385 | 4.18E-01 | - |
| rs7948646 | 11 | *DLG2* | A | 0.354 | 0.203 | **1.14E-03** | **2.14[1.35-3.41]** | 0.321 | 0.312 | 6.29E-01 | - |
| rs542892 | 11 | *DLG2* | G | 0.424 | 0.258 | **6.71E-04** | **2.12[1.37-3.27]** | 0.393 | 0.404 | 9.49E-01 | - |
| rs290186 | 11 | *CCDC89* | A | 0.753 | 0.604 | **1.96E-03** | **1.99[1.28-3.09]** | 0.704 | 0.689 | 6.68E-01 | - |
| rs2187132 | 11 | *CASP12* (265 kb) | A | 0.859 | 0.720 | **8.66E-04** | **2.36[1.41-3.95]** | 0.765 | 0.803 | 1.93E-01 | - |
| rs472826 | 11 | *SLC35F2* (38 kb) | T | 0.677 | 0.511 | **1.08E-03** | **2.00[1.32-3.04]** | 0.625 | 0.593 | 5.28E-01 | - |
| rs10791126 | 11 | *NTM* (208 kb) | T | 0.798 | 0.637 | **4.88E-04** | **2.25[1.42-3.56]** | 0.699 | 0.668 | 3.48E-01 | - |
| rs7305157 | 12 | *BCAT1* (42 kb) | C | 0.162 | 0.071 | **6.56E-03** | **2.51[1.27-4.94]** | 0.133 | 0.134 | 9.26E-01 | - |
| rs1027949 | 12 | *GIT2* | G | 0.222 | 0.099 | **1.15E-03** | **2.60[1.44-4.70]** | 0.184 | 0.207 | 4.21E-01 | - |
| rs668001 | 13 | *ATP8A2* | T | 0.392 | 0.233 | **1.28E-03** | **2.12[1.34-3.37]** | 0.301 | 0.332 | 4.58E-01 | - |
| rs7990079 | 13 | *LHFP* | T | 0.500 | 0.407 | 6.78E-02 | - | - | - | - | - |
| rs2274758 | 13 | *TBC1D4* | T | 0.636 | 0.445 | **1.83E-04** | **2.18[1.45-3.29]** | 0.592 | 0.61 | 5.60E-01 | - |
| rs1469600 | 13 | *TBC1D4* | T | 0.628 | 0.438 | **2.45E-04** | **2.16[1.43-3.27]** | 0.598 | 0.605 | 9.70E-01 | - |
| rs1522012 | 13 | *SLITRK5* (707 kb) | A | 0.788 | 0.659 | **4.99E-03** | **1.92[1.21-3.03]** | 0.816 | 0.766 | 1.16E-01 | - |
| rs7157956 | 14 | *SMOC1* | C | 0.545 | 0.401 | **4.88E-03** | **1.79[1.19-2.69]** | 0.480 | 0.484 | 8.45E-01 | - |
| rs7148202 | 14 | *ENTPD5* | T | 0.556 | 0.385 | **8.56E-04** | **2.00[1.33-3.01]** | 0.485 | 0.472 | 7.08E-01 | - |
| rs8028364 | 15 | *IL16* | G | 0.833 | 0.670 | **2.22E-04** | **2.46[1.51-3.99]** | 0.704 | 0.747 | 2.53E-01 | - |
| rs11072994 | 15 | *IL16* | T | 0.838 | 0.703 | **1.67E-03** | **2.19[1.33-3.59]** | 0.724 | 0.766 | 2.60E-01 | - |
| rs17516032 | 15 | *SV2B* | A | 0.712 | 0.511 | **5.65E-05** | **2.37[1.55-3.61]** | 0.612 | 0.595 | 6.55E-01 | - |
| rs7224497 | 17 | *OR3A4P* | C | 0.192 | 0.071 | **5.77E-04** | **3.09[1.59-6.01]** | 0.102 | 0.127 | 4.38E-01 | - |
| rs9911870 | 17 | *OR3A4P* | G | 0.192 | 0.071 | **5.77E-04** | **3.09[1.59-6.01]** | 0.102 | 0.128 | 4.05E-01 | - |
| rs11658522 | 17 | *INTS2* | G | 0.332 | 0.170 | **3.18E-04** | **2.42[1.48-3.94]** | 0.289 | 0.243 | 2.33E-01 | - |
| rs1005318 | 17 | *CASC17* (85 kb) | A | 0.825 | 0.631 | **2.54E-05** | **2.76[1.70-4.45]** | 0.704 | 0.701 | 9.56E-01 | - |
| rs17225585 | 17 | *CASC17* (172 kb) | T | 0.157 | 0.060 | **2.83E-03** | **2.89[1.40-5.93]** | 0.143 | 0.107 | 1.11E-01 | - |
| rs652264 | 18 | *DLGAP1* | A | 0.636 | 0.467 | **9.06E-04** | **2.00[1.32-3.01]** | 0.628 | 0.592 | 3.92E-01 | - |
| rs17767647 | 18 | *KC6* (378 kb) | T | 0.904 | 0.769 | **2.83E-04** | **2.83[1.57-5.07]** | 0.842 | 0.815 | 3.88E-01 | - |
| rs9965625 | 18 | *TCF4* (59 kb) | A | 0.403 | 0.270 | **8.35E-03** | **1.83[1.16-2.87]** | 0.356 | 0.335 | 5.44E-01 | - |
| rs2826880 | 21 | *NCAM2* | G | 0.641 | 0.435 | **1.00E-04** | **2.33[1.52-3.57]** | 0.532 | 0.525 | 9.43E-01 | - |
| rs2837636 | 21 | *DSCAM* | T | 0.677 | 0.527 | **2.94E-03** | **1.88[1.24-2.84]** | 0.561 | 0.629 | 1.04E-01 | - |

Nominally significant *P*-values (*P*≤5.00E-02) and their respective odd ratios (OR) and 95% confidence intervals (CI) are highlighted in bold. Only SNPs associated in the discovery dataset (100 IA cases and 92 controls) were tested for association in the replication dataset (100 IA cases and 407 controls).

Chr.: Chromosome; kb: Kilobase pairs; F_cases_ and F_controls_: Allele frequency in cases and controls, respectively; *P*: *P*-value in the allelic chi-square test.
