# Supplementary material for: Genetic Variants Underlying Risk of Intracranial Aneurysms: Insights from a GWAS in Portugal
Source: PLoS One. 2015 Jul 17;10(7):e0133422. doi: 10.1371/journal.pone.0133422 (PMC4505843; doi:10.1371/journal.pone.0133422)
Supplement: S4 Table — (DOCX) [file pone.0133422.s006.docx]

**S4 Table. |RAS_diff_| of single nucleotide polymorphisms (SNPs) GWAS-associated with intracranial aneurysms (IA) before December 2014**

| **Reference** | **SNP** | **Proxy SNP** | **r^2^** | **\|RAS_diff_\|** |
| --- | --- | --- | --- | --- |
| Bilguvar et al. [9] | rs700651 | rs10931791 | 0.96 | 4.8% |
|  | rs10958409 | rs7845242 | 0.89 | 0.8% |
| Yasuno et al. [10] | rs9298506 |  |  | 4.2% |
|  | rs12413409 |  |  | 6.6% |
| Akiyama et al. [12] | rs1930095 |  |  | 6.2% |
|  | rs4628172 | rs6461180 | 1.00 | 1.1% |
|  | rs7781293 | rs10261081 | 1.00 | 1.9% |
|  | rs7550260 | rs7542311 | 1.00 | 3.0% |
|  | rs9864101 | rs903471 | 0.96 | 7.7% |
| Low et al. [13] | rs6842241 | rs17612742 | 1.00 | 0.7% |
|  | rs10757272 |  |  | 8.2% |
| Foroud et al. [14] | rs6475606 |  |  | 7.6% |
|  | rs1072737 |  |  | 2.8% |
| Foroud et al. [15] | rs10230207 |  |  | 1.4% |
| Kurki et al. [18] | rs74972714 | NA |  |  |
|  | rs919433 | rs1560277 | 1.00 | 1.8% |
|  | rs113816216 | NA |  |  |
|  | rs75018213 | NA |  |  |
|  | rs1333042 |  |  | 8.4% |

If the associated SNP was not included in the Affymetrix Human SNP Array 6.0 used in the current study, the proxy SNP in the microarray with the strongest linkage disequilibrium (as measured by r^2^ in the HapMap CEU samples) is indicated.

GWAS: genome-wide association study; |RAS_diff_|: Absolute value of the relative allele score difference; NA: Not available.
