# Supplementary material for: Genetic Variants Underlying Risk of Intracranial Aneurysms: Insights from a GWAS in Portugal
Source: PLoS One. 2015 Jul 17;10(7):e0133422. doi: 10.1371/journal.pone.0133422 (PMC4505843; doi:10.1371/journal.pone.0133422)
Supplement: S5 Table — (DOCX) [file pone.0133422.s007.docx]

**S5 Table. Top findings of genome-wide association studies for intracranial aneurysms**

| **Study reference** | **Discovery dataset** | **Replication dataset** | **IA-associated SNP** | **Position** | **Candidate gene(s)** | **OR[95% CI]** | ***P*** |
| --- | --- | --- | --- | --- | --- | --- | --- |
| Bilguvar et al. [9] | 920 Cases, | 494 Cases, | rs700651 | 2q33.1 | *BOLL, PLCL1* | 1.24[1.15-1.34] | 4.40E-08 |
|  | 985 Controls | 676 Controls | rs10958409 | 8q11.23 | *SOX17* | 1.36[1.24-1.49] | 1.40E-10 |
|  | (Finnish) | (Japanese) | rs1333040 | 9p21.3 | *CDKN2BAS* | 1.29[1.19-1.40] | 1.40E-10 |
|  | 781 Cases, |  |  |  |  |  |  |
|  | 6424 Controls |  |  |  |  |  |  |
|  | (Dutch) |  |  |  |  |  |  |
| Yasuno et al. [10]* | 808 Cases, | 3111 Cases, | rs9298506 | 8q12.1 | *SOX17* | 1.28[1.20-1.38] | 1.30E-12 |
|  | 4393 Controls | 1666 Controls | rs1333040 | 9p21.3 | *CDKN2BAS* | 1.32[1.25-1.39] | 1.50E-22 |
|  | (Finnish) | (Japanese) | rs12413409 | 10q24.32 | *CNNM2* | 1.29[1.19-1.40] | 1.20E-09 |
|  | 1972 Cases, |  | rs9315204 | 13q13.1 | *STARD13* | 1.20[1.13-1.28] | 2.50E-09 |
|  | 8122 Controls |  | rs11661542 | 18q11.2 | *RBBP8* | 1.22[1.15-1.28] | 1.10E-12 |
|  | (Other European) |  |  |  |  |  |  |
| Akiyama et al. [12] | 288 Cases, | 739 Cases, | rs7550260 | 1q21 | *ARHGEF11* | 1.32[1.15-1.50] | 4.93E-05 |
|  | 194 Controls | 659 Controls | rs9864101 | 3p25.2 | *IQSEC1* | 1.49[1.23-1.80] | 3.63E-05 |
|  | (Japanese) | (Japanese) | rs7781293 | 7p21.2 | *TMEM195* | 1.32[1.16-1.50] | 2.78E-05 |
|  |  |  | rs4628172 | 7p21.2 | *TMEM195* | 1.30[1.14-1.48] | 1.32E-05 |
|  |  |  | rs1930095 | 9q31.2-31.3 | Intergenic region | 1.44[1.22-1.71] | 1.31E-05 |
| Low et al. [13] | 1383 Cases, | 1048 Cases, | rs6842241 | 4q31.22 | *EDNRA* | 1.25[1.16-1.34] | 9.58E-09 |
|  | 5484 Controls | 7212 Controls | rs10757272 | 9p21.3 | *CDKN2BAS* | 1.21[1.13-1.30] | 1.55E-07 |
|  | (Japanese) | (Japanese) | rs1333040 | 9p21.3 | *CDKN2BAS* | 1.16[1.09-1.25] | 5.56E-05 |
|  |  |  | rs671 | 12q24.12 | *ALDH2* | 1.24[1.15-1.34] | 2.63E-06 |
| Foroud et al. [14] | 1483 Cases, | NA | rs1072737 | 8q11.23 | *SOX17* | 1.25[NA] | 8.70E-05 |
|  | 1683 Controls |  | rs6475606 | 9p21.3 | *CDKN2BAS* | 1.35[NA] | 3.60E-08 |
|  | (White US residents) |  |  |  |  |  |  |
| Foroud et al. [15]** | 2617 Cases, | 717 Cases, | rs10230207 | 7p21.1 | *HDAC9* | 1.21[1.14-1.28] | 9.91E-10 |
|  | 2548 Controls | 3004 Controls |  |  |  |  |  |
|  | (White European) | (Dutch) |  |  |  |  |  |
|  |  | 799 Cases, |  |  |  |  |  |
|  |  | 2317 Controls |  |  |  |  |  |
|  |  | (Finnish) |  |  |  |  |  |
| Kurki et al. [18] | 760 cases, | 858 Cases, | rs74972714 | 2q23.3 | *LYPD6* | 1.89[NA] | 1.42E-09 |
|  | 2513 Controls | 4048 Controls | rs919433 | 2q33.1 | *ANKRD44* | 1.27[NA] | 2.20E-12 |
|  | (Finnish) | (Finnish) | rs113816216 | 5q31.3 | *FSTL4* | 1.66[NA] | 3.17E-08 |
|  |  | 717 Cases, | rs75018213 | 6q24.2 | *EPM2A* | 1.87[NA] | 7.14E-11 |
|  |  | 3004 Controls | rs1333042 | 9p21.3 | *CDKN2BAS* | 1.31[NA] | 6.71E-16 |
|  |  | (Dutch) |  |  |  |  |  |
| Present report | 100 Cases, | 100 Cases, | rs4667622 | 2q31.1 | *UBR3 - MYO3B* | 1.75[1.33-2.33] | 4.00E-05 |
|  | 92 Controls | 407 Controls | rs6599001 | 3p22.2 | *SCN11A - WDR48* | 0.50[0.35-0.72] | 2.20E-04 |
|  | (Portuguese) | (Portuguese) | rs3932338 | 5p14.2 | *PRDM9* | 0.63[0.48-0.84] | 1.29E-03 |
|  |  |  | rs10943471 | 6q14.1 | *HTR1B* | 0.55[0.40-0.76] | 3.21E-04 |

*Follow-up study of Bilguvar et al. [9]. **Follow-up study of Foroud et al. [14].

Odds ratios (OR) and 95% confidence intervals (CI) are relative to the allele on the forward strand of the human genome reference sequence.

IA: Intracranial aneurysm; SNP: Single nucleotide polymorphism; *P*: *P*-values from meta-analysis; NA: Not available.
